# Dual-specificity kinase DYRK3 phosphorylates p62 at the Thr-269 residue and promotes melanoma progression

**DOI:** 10.1016/j.jbc.2024.107206

**Published:** 2024-03-20

**Authors:** Ye Hyung Lee, A-Rum Yoon, Chae-Ok Yun, Kwang Chul Chung

**Affiliations:** 1 Department of Systems Biology, College of Life Science and Biotechnology, Yonsei University, Seoul, South Korea; 2 Department of Bioengineering, College of Engineering, Hanyang University, Seoul, South Korea

**Keywords:** DYRK3, p62, phosphorylation, TRAF6, mTORC1, melanoma

## Abstract

Melanoma is a type of skin cancer that originates in melanin-producing melanocytes. It is considered a multifactorial disease caused by both genetic and environmental factors, such as UV radiation. Dual-specificity tyrosine-phosphorylation-regulated kinase (DYRK) phosphorylates many substrates involved in signaling pathways, cell survival, cell cycle control, differentiation, and neuronal development. However, little is known about the cellular function of DYRK3, one of the five members of the DYRK family. Interestingly, it was observed that the expression of DYRK3, as well as p62 (a multifunctional signaling protein), is highly enhanced in most melanoma cell lines. This study aimed to investigate whether DYRK3 interacts with p62, and how this affects melanoma progression, particularly in melanoma cell lines. We found that DYRK3 directly phosphorylates p62 at the Ser-207 and Thr-269 residue. Phosphorylation at Thr-269 of p62 by DYRK3 increased the interaction of p62 with tumor necrosis factor receptor–associated factor 6 (TRAF6), an already known activator of mammalian target of rapamycin complex 1 (mTORC1) in the mTOR-involved signaling pathways. Moreover, the phosphorylation of p62 at Thr-269 promoted the activation of mTORC1. We also found that DYRK3-mediated phosphorylation of p62 at Thr-269 enhanced the growth of melanoma cell lines and melanoma progression. Conversely, *DYRK3* knockdown or blockade of p62-T269 phosphorylation inhibited melanoma growth, colony formation, and cell migration. In conclusion, we demonstrated that DYRK3 phosphorylates p62, positively modulating the p62-TRAF6-mTORC1 pathway in melanoma cells. This finding suggests that DYRK3 suppression may be a novel therapy for preventing melanoma progression by regulating the mTORC1 pathway.

A family of dual-specificity tyrosine-(Y)-phosphorylation-regulated kinases (DYRKs), including DYRK1A, DYRK1B, DYRK2, DYRK3, and DYRK4, commonly mediate autophosphorylation of tyrosine residues and the phosphorylation of serine/threonine residues on a number of substrates (1, 2). These DYRK members have similar structures and conserved catalytic domains, but each individual member has distinct N-terminal and C-terminal regions responsible for substrate specificity and differential intrinsic functions in cancer and neuronal cells. Among them, DYRK3 has both an oncogenic and tumor suppressor role, depending on the phosphorylation targets (3). For example, DYRK3 directly phosphorylates PRAS40, the mammalian target of rapamycin complex 1 (mTORC1) inhibitor, at the Thr-246 residue, releasing mTORC1 from stress granules to downstream signaling pathways and promoting mTORC1 (4). Additionally, the expression of DYRK3 is highly increased in neuroblastoma and closely associated with decreased survival in neuroblastoma patients (5). Moreover, DYRK3 phosphorylates and activates SIRT1, promoting cell survival against toxic stress (6).

mTORC1 is a central kinase complex that plays an essential role in cellular growth, protein synthesis, and autophagy. Sequestosome-1 (SQSTM1, p62) acts as an autophagy adaptor and binds to Raptor, an integral component of the mTORC1 pathway, as well as tumor necrosis factor receptor–associated factor 6 (TRAF6), an ubiquitin E3 ligase. It is required for the translocation of mTORC1 to the lysosome (7). TRAF6 is then recruited to and activates mTORC1 through p62 in nutrient-stimulated cells. Additionally, TRAF6 is necessary for the translocation of mTORC1 to the lysosomes, and the TRAF6-catalyzed K63 ubiquitination of mTOR regulates mTORC1 activation by nutrients. TRAF6, through its interaction with p62 and activation of mTORC1, modulates autophagy and serves as an important mediator in cancer cell proliferation.

p62 is an ubiquitin-binding protein involved in cell signaling, oxidative stress, and autophagy. It recognizes ubiquitinated cargos through its C-terminal ubiquitin-associated (UBA) domain and attracts them to the autophagosome membrane via the microtubule-associated protein 1 light chain 3-interacting region (LIR) (8). In addition to the UBA and LIR domains, p62 contains other structural domains, including the N-terminal Phox1 and Bem1p (PB1) domain, ZZ-type zinc finger (ZZ) domain, TRAF6-binding (TB) domain, and Kelch-like ECH-associated protein 1 (Keap1)-interacting region (9). The region between the TB domain and ZZ domain recruits regulatory-associated protein of mTOR or Raptor, ultimately activating mTORC1 (10). Furthermore, serving as a signaling hub, p62 interacts with various binding partners to mediate multiple cellular functions, including apoptosis and activation of mTORC1, NF-κB, or Nrf2 (10, 11, 12, 13).

Melanoma is one of the most malignant skin tumors, with a rapidly increasing incidence worldwide. Recently, melanoma has been defined as a multifactorial disease arising from an interaction between genetic susceptibility and environmental exposure. The most significant environmental risk factor for promoting malignant melanoma is exposure to UV rays, which have a genotoxic effect (14). Additionally, *CDKN2A* and *CDK4* are two high-risk susceptibility genes for cutaneous malignant melanoma (15).

While the tumor-modulatory role of DYRK3 has been demonstrated in a limited number of recent studies, the detailed mechanisms underlying how DYRK3 affects the development of various types of cancers and its downstream substrates have not been extensively investigated. Interestingly, our preliminary analyses of several cancer databases have revealed strong expression of both DYRK3 and p62 in melanoma cell lines. Since the biochemical and functional relationship between these two proteins has not been elucidated yet, the present study aims to further investigate how these two proteins contribute to the progression of melanoma tumors. Our data suggest that DYRK3 directly phosphorylates p62 at Thr-269, stimulating the activation of the mTORC1-mediated downstream signaling pathway and ultimately leading to an increase in the oncogenic function of p62 in melanoma.

## Results

### The expression levels of DYRK3 and p62 were higher in melanoma cells compared to other cell lines

**Figure 1:**
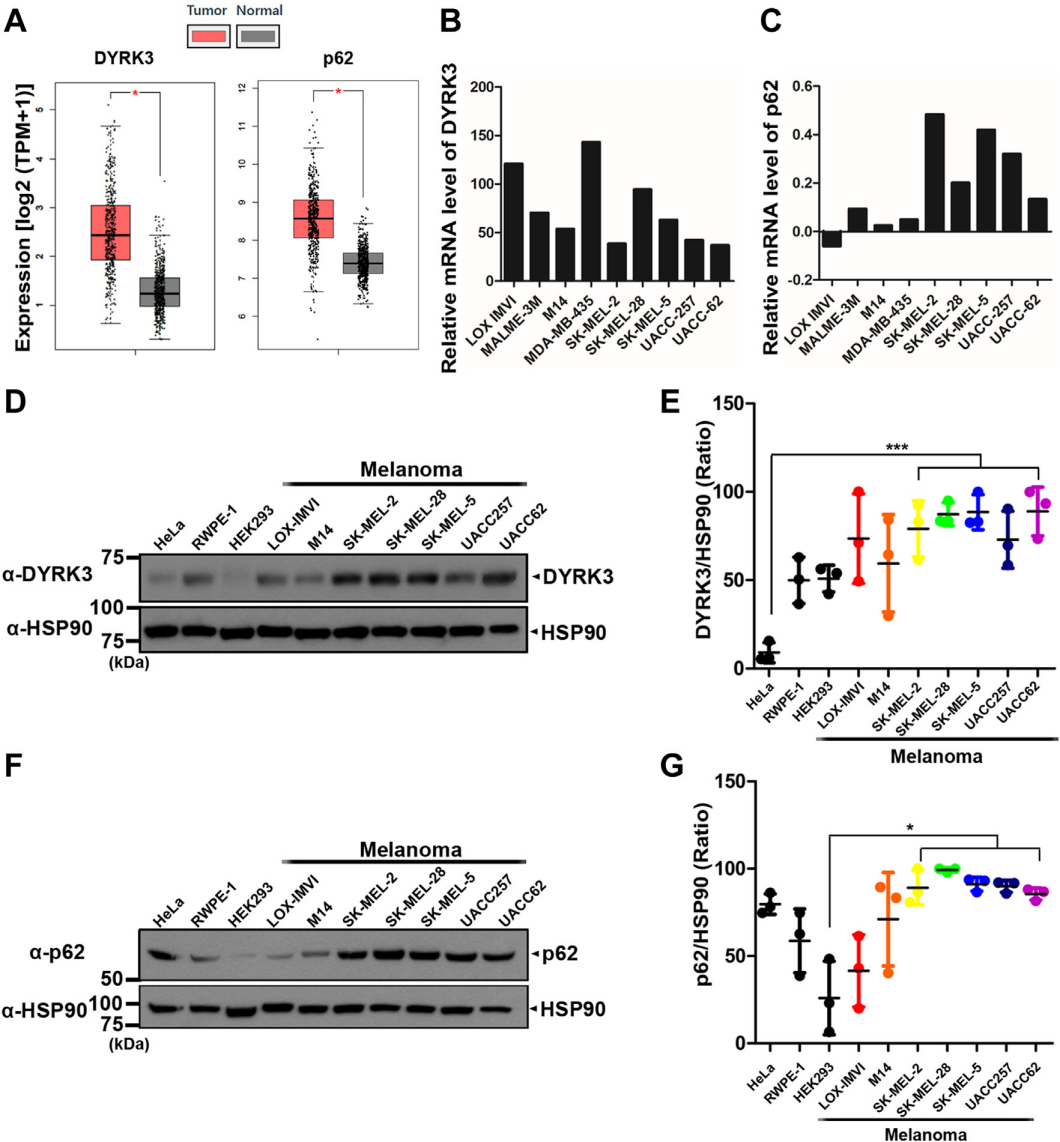
**The expression of DYRK3 and p62 was higher in various melanoma cells lines.***A*, the median expression level of the *DYRK3* and *p62* genes in normal and melanoma cancer tissues was analyzed by GEPIA (TPM: Transcripts per Million). According to TCGA data, the analysis indicated that the expression level of DYRK3 and p62 in melanoma cancer tissues (n = 461) was enhanced compared to normal tissues (n = 558). GEPIA, Gene Expression Profiling Interaction Analysis (http://gepia.cancer-pku.cn). *B* and *C*, microarray data of DYRK3 and p62 from the U.S. National Cancer Institute (http://dtp.nci.nih.gov/mtweb/targetdata). The expression of DYRK3 (Exp. ID: 30355; https://dtp.cancer.gov/mtweb/targetinfo?moltid=GC30356&moltnbr=30355) and p62 (SQSTM1, Exp. ID: 9233; https://dtp.cancer.gov/mtweb/targetinfo?moltid=GC18635&moltnbr=9233) is consistently higher in melanoma cell lines than in the control. *D*, immunoblot analyses for DYRK3 levels in three control cell lines (HeLa, RWPE-1, and HEK293) and seven melanoma cancer cells were shown. *E*, quantification of DYRK3 expression levels from the blots in (*D*). All data represent the mean ± standard deviation of three independent experiments (∗∗∗*p* < 0.001). *F*, immunoblot analyses for p62 levels in three control cell lines (HeLa, RWPE-1, and HEK293) and seven melanoma cancer cells were shown. *G*, quantification of p62 expression levels from the blots in (*D*). All data represent the mean ± standard deviation of three independent experiments (∗*p* < 0.05). DYRK, dual-specificity tyrosine-phosphorylation-regulated kinase; HEK, human embryonic kidney cells; TCGA, The Cancer Genome Atlas.

Considering the previous report that DYRK3 regulates mTORC1 signaling through the modulation dynamin-related protein 1 (DRP-1) under radiation stress, consequently promoting glioblastoma progression (3), it could be hypothesized that DYRK3 also has an oncogenic feature in other types of cancer, including melanoma skin cancer. To further verify this hypothesis and, if that is the case, to identify additional phosphorylation target(s) of DYRK3, we first analyzed the relationship between the expression of DYRK3 and various cancer types. For this trial, we employed the Gene Expression Profiling Interactive Analyses, a web server of cancer data bases. According to the analysis of The Cancer Genome Atlas data through Gene Expression Profiling Interactive Analyses, the expression level of DYRK3 was significantly increased in melanoma cancer tissues compared with normal skin tissues (Fig. 1*A*). The expression level of p62 was also elevated in melanoma cancer tissues compared to normal skin tissues (Fig. 1*A*), consistent with the previous report that p62 is overexpressed during melanoma progression (10). Furthermore, the analysis of microarray data from the U.S. National Cancer Institute database demonstrated an increase in *DYRK3* mRNA level in multiple melanoma cancer cell lines. Similarly, *p62* mRNA level was also enhanced in many melanoma cancer cell lines (Fig. 1, *B* and *C*).

Next, we examined whether the intracellular DYRK3 protein level could be increased in various melanoma cell lines (Fig. 1, *D* and *E*). Among the different cancer cell lines tested, including human cervical carcinoma cells (HeLa), human embryonic kidney cells (HEK), human prostate cells (RWPE-1), and melanoma cell lines (SK-Mel-28, SK-Mel-5, UACC257, and UACC62), the highest expression of DYRK3 was observed in melanoma cell lines. Additionally, p62 was found to be highly expressed in SK-Mel-2, SK-Mel-28, SK-Mel-5, UACC257, and UACC62cells among the melanoma cell lines (Fig. 1, *F* and *G*). Taken together, these results indicate that the expression levels of DYRK3 and p62 are higher in melanoma cells than other cancer cell lines, suggesting that DYRK3 and p62 may play a role in the progression of melanoma.

### DYRK3 interacts with p62

**Figure 2:**
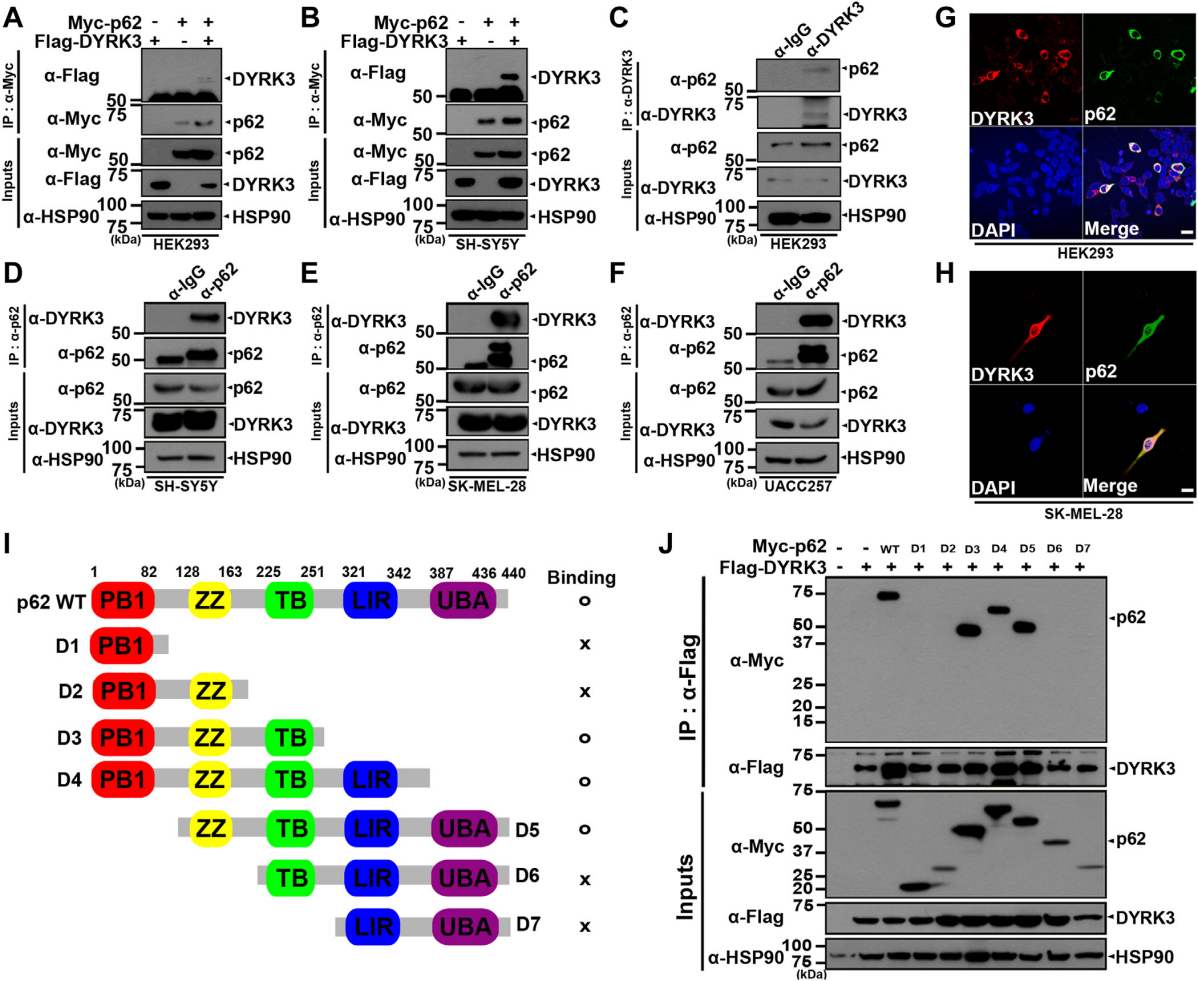
**DYRK3 interacts with p62.***A* and *B*, HEK293 (*A*) or SH-SY5Y cells (*B*) were transfected for 24 h with a plasmid encoding Myc-p62 or Flag-DYRK3 alone or in combination. Cell lysates were immunoprecipitated with an anti-Myc antibody, followed by immunoblotting with the indicated antibody. Hsp90 served as a loading control. *C*–*F*, where indicated, cell lysates prepared from HEK293 (*C*), SH-SY5Y (*D*), SK-Mel-28 (*E*), or UACC257 (*F*) cells were immunoprecipitated with anti-DYRK3, anti-p62, or preimmune IgG, followed by immunoblotting with the indicated antibody. *G* and *H*, representative confocal images of immunostaining of HEK293 (*G*) or SK-Mel-28 cells (*H*) stably expressing both Flag-DYRK3 (*red*) and Myc-p62 (*green*). Nuclei were counterstained with DAPI (*blue*). The scale bar represents 20 μm. *I*, the diagram outlines WT p62 (p62-WT) and its deletion mutants, delineating various domains such as the N-terminal Phox1 and Bem1p (PB1) domain, zinc finger (ZZ) domain, tumor necrosis factor receptor-associated factor 6 (TRAF6)-binding (TB) motif, LC3-interacting region (LIR) domain, and the C-terminal ubiquitin-associated (UBA) domain. Summarized results from co-IP assays exploring DYRK3 and p62 interaction are displayed on the *left* and *right*, with 'x' indicating no binding and 'o' indicating binding. *J*, HEK293 cells were transfected for 24 h with a plasmid encoding Myc-tagged p62-WT, one of its deletion mutants, or Flag-DYRK3 alone or in combination. Cell lysates were immunoprecipitated with an anti-Myc antibody, followed by immunoblotting with the indicated antibody. Hsp90 served as a loading control. DAPI, 4′,6-diamidino-2-phenylindole; DYRK, dual-specificity tyrosine-phosphorylation-regulated kinase; HEK, human embryonic kidney cells; co-IP, coimmunoprecipitation.

We then examined whether there would be a biochemical and/or functional interaction between DYRK3 and p62 in mammalian cells, and if so, how they might affect melanoma progression. HEK293 cells were transiently transfected with Myc-p62 or/and Flag-DYRK3, and cell lysates were immunoprecipitated with anti-Myc antibody. Immunoblot analysis of the anti-Myc immunocomplexes with anti-Flag antibodies revealed that DYRK3 binds to p62 (Fig. 2*A*). The same coimmunoprecipitation (co-IP) experiment was performed using human neuroblastoma SH-SY5Y cells, and immunoblot analysis of the anti-Myc immunocomplexes with anti-Flag antibodies also showed that DYRK3 binds to p62 (Fig. 2*B*). Furthermore, cell lysates from SH-SY5Y or HEK293 cells were immunoprecipitated with either preimmune immunoglobulin G (IgG), anti-DYRK3, or anti-p62 antibodies. Immunoblot analyses of the immunocomplex samples using DYRK3 or p62 antibody revealed that endogenous DYRK3 interacts with endogenous p62 (Fig. 2, *C* and *D*). These results indicate that the interaction between DYRK and p62 is not an artifact of DNA transfection but rather a specific interaction in mammalian cells. Moreover, we examined whether there is an interaction between DYRK3 and p62 in several melanoma cells and found that endogenous DYRK3 binds to endogenous p62 in melanoma SK-Mel-28 and UACC257 cell lines (Fig. 2, *E* and *F*). To determine whether DYRK3 and p62 are colocalized within the cell, HEK293 or SK-Mel-28 cells were transfected with Flag-DYRK3 or/and Myc-p62. Immunostaining of the cells with anti-Flag (red) or anti-Myc (green) antibodies revealed that both proteins are primarily expressed in the cytoplasm, but there are also small amounts present in the nucleus (Fig. 2, *G* and *H*). Overall, these findings suggest that DYRK3 interacts with p62 in mammalian system and melanoma cancer cells.

As described previously and shown in Figure 2*I*, the p62 protein contains multiple conserved functional and structural domains. Specifically, the N-terminal PB1 domain is involved in the self-oligomerization of p62, serving as an interaction surface for other PB1-containing proteins such as atypical protein kinase C (16). In addition, the central zinc finger ZZ and TB domains interact with the receptor-interacting proteins and TRAF6 proteins, respectively, positively regulating the downstream pathway of NF-*κ*B (17, 18). Lastly, the LIR and the C-terminal UBA domain of p62 links the autophagic machinery to ubiquitinated substrates to promote the selective degradation of these molecules.

Next, we determined which domain(s) of p62 is important for DYRK3 binding. For this assay, seven deletion mutants of p62 (D1 ∼ D7) lacking or partly possessing those domains, such as the PB1, ZZ, TB, LIR, and the UBA region (Fig. 2*I*) were used. After WT p62 or one of those mutants was coexpressed with DYRK3 in HEK293 cells, their interaction was assessed by co-IP analysis. As shown in Figure 2*J*, co-IP analyses of cell lysates revealed that three deletion mutants of p62 (D3, D4, and D5), as well as WT p62, bind well to DYRK3. However, the other four mutants of p62, such as D1, D2, D6, or D7, exhibited greatly reduced binding to DYRK3 compared to WT p62 (Fig. 2*J*). These results suggest that DYRK3 binds to the 128∼251 amino acid region of p62, which spans the central ZZ and TB domains.

### DYRK3 phosphorylates p62 at S207 and T269 in the TB domain

**Figure 3:**
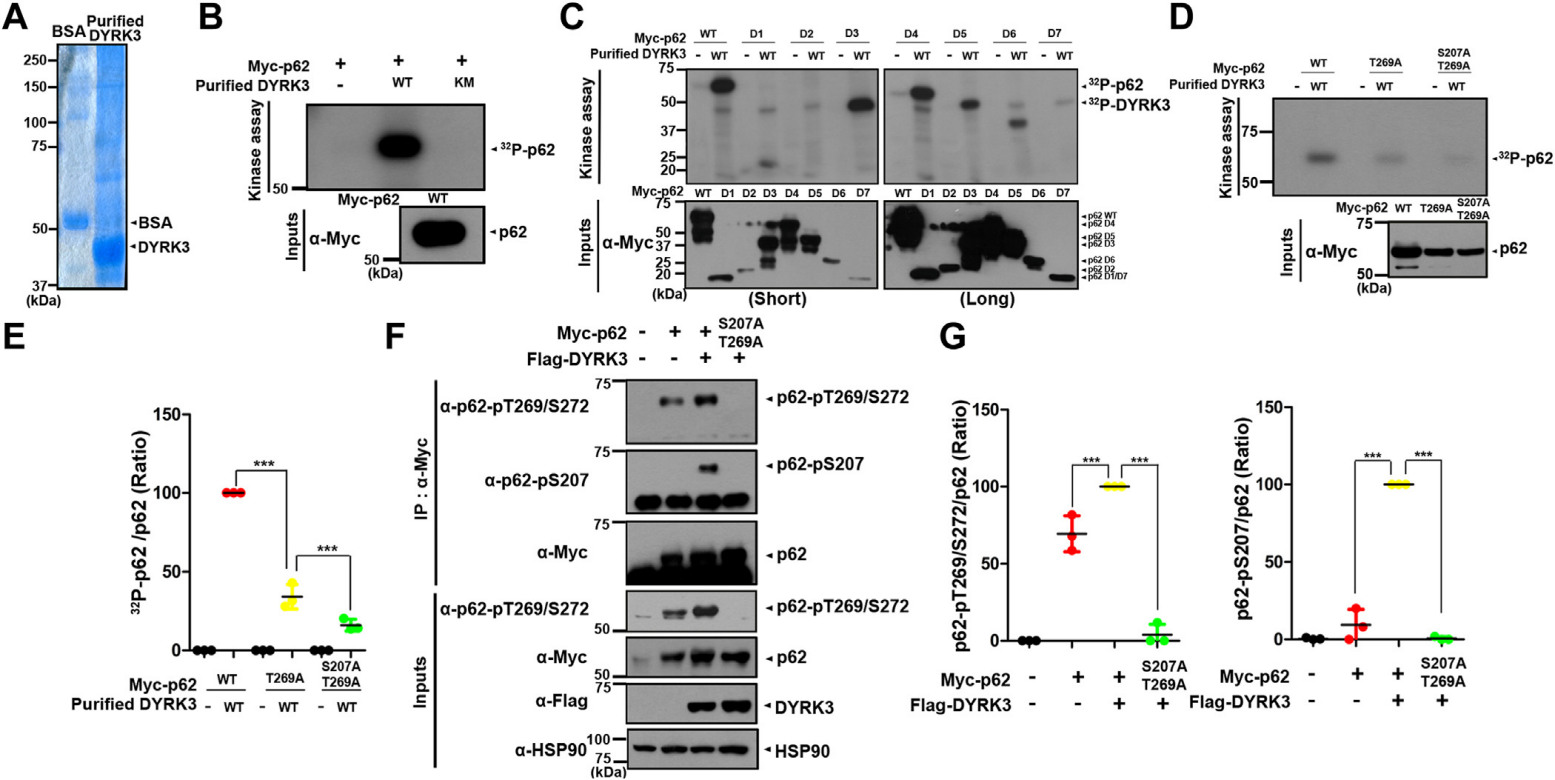
**DYRK3 phosphorylates p62 at S207 and T269 residues in the TB domain.***A*, recombinant purified DYRK3 protein and commercial bovine serum albumin (BSA) as a control were stained with Coomassie blue dyes. *B*, after HEK293 cells were transfected for 24 h with a plasmid encoding Myc-p62, cell lysates (∼1000 μg of protein) were immunoprecipitated with an anti-Myc antibody. Where specified, the samples were mixed with bacterially expressed WT DYRK3 (DYRK3-WT) or its kinase-inactive mutant (DYRK3-KM), incubated for 30 min at 30 ^°^C with the kinase buffer and [γ-^32^P]ATP, resolved by SDS-PAGE, and analyzed by autoradiography. Proper expression of transiently expressed p62 in cell extracts was verified by immunoblotting with an anti-Myc antibody (Input). *C*, HEK293 cells were transfected for 24 h with a plasmid encoding Myc-p62-WT or one of its deletion mutants (D1-D7), followed by immunoprecipitation with an anti-Myc antibody. The anti-Myc immunocomplexes as a substrate were mixed with bacterially expressed DYRK3-WT, incubated for 30 min at 30 ^°^C with the kinase buffer and [γ-^32^P]ATP, resolved by SDS-PAGE, and analyzed by autoradiography. *D*, HEK293 cells were transfected for 24 h with a plasmid encoding Myc-p62-WT, Myc-p62-T269A, or Myc-p62-S207/T269A, followed by immunoprecipitation with an anti-Myc antibody. The anti-Myc immunocomplexes as a substrate were mixed with bacterially expressed DYRK3-WT, incubated for 30 min at 30 ^°^C with the kinase buffer and [γ-^32^P]ATP, resolved by SDS-PAGE, and analyzed by autoradiography. *E*, quantification of the protein band densities in (*D*) was performed. All data represent the mean ± standard deviation of three independent experiments (∗∗∗*p* < 0.001). *F*, HEK293 cells were transfected for 24 h with a plasmid encoding Myc-p62-WT, Myc-p62-S207/T269A, or Flag-DYRK3 alone or in combination. Cell lysates were immunoprecipitated with an anti-Myc antibody, followed by immunoblotting with anti-pT269/S272-p62 or anti-pS207-p62 antibodies. *G*, quantification of the protein band densities in (*F*) was performed. All data represent the mean ± standard deviation of three independent experiments (∗∗∗*p* < 0.001). DYRK, dual-specificity tyrosine-phosphorylation-regulated kinase; HEK, human embryonic kidney cells; TB, TRAF6-binding.

To gain further insight into the link between DYRK3 and p62, we examined whether DYRK3 directly phosphorylates p62. To this end, we used an *in vitro* kinase assay (Fig. 3, *A*–*E*). For this assay, bacterial vectors expressing His-tagged WT DYRK3 (DYRK3-WT) or its kinase-inactive mutant with the substitution of K238M (DYRK3-KM) were expressed in *Escherichia coli*, and their recombinant proteins were purified and used as kinases (Fig. 3*A*). HEK293 cells were transfected with a mammalian plasmid encoding Myc-p62 to provide a substrate, and the anti-Myc immunocomplexes were prepared. After incubating the anti-Myc-p62 immunoprecipitates with [γ-^32^P]ATP alone or with either bacterially purified recombinant DYRK3-WT or DYRK3-KM, the *in vitro* kinase assays for phosphorylation revealed that WT DYRK3 phosphorylates p62, whereas this effect was not observed with DYRK3-KM (Fig. 3*B*). These results suggested that DYRK3 directly phosphorylates p62.

Next, we attempted to identify the specific site(s) in p62 targeted by DYRK3. To achieve this, we initially investigated which domain(s) of p62 could be phosphorylated by DYRK3. *In vitro* kinase assays revealed that four deletion mutants of p62 (D3, D4, D5, and D6), all containing the TB domain, were significantly phosphorylated by DYRK3 compared to the WT p62. However, we did not observe this noticeable modification by DYRK3 in the D1, D2, and D7 mutants of 62 (Fig. 3*C*). These results indicate that DYRK3 specifically phosphorylates p62 at site(s) located within the TB domain.

DYRK3 is known to undergo tyrosine autophosphorylation and catalyze the phosphorylation of substrates on serine/threonine residues. The TB domain of p62 contains a total of 21 serine (S) and threonine (T) residues. Based on this report and using the p62-D6 mutant as a template, total 11 p62 mutants were generated. These mutants had single, double, or triple point mutations of the S/T sites with alanine (Ala). After transfecting HEK293 cells with the plasmid encoding Myc-tagged p62-D6 mutant or one of the D6-derived 11 mutants, anti-Myc immunoprecipitates were prepared as a substrate. An *in vitro* kinase assay was then conducted using anti-Myc immunocomplexes of p62 as a substrate alone or together with bacterial recombinant DYRK3-WT protein as a kinase. Measuring the relative phosphorylation status of these p62 mutants revealed that the p62-D6-S266/T269/S272A mutant displayed significantly reduced phosphorylation than that of p62-D7, which served as a negative control (Fig. S1*A*). However, the amount of phosphorylation in the p62-D6-S266/T269/S272A triple-mutant was not completely blocked by DYRK3, indicating the presence of an additional phosphorylation site outside those three residues.

In the subsequent trial, we used the p62-D5 mutant as a template due to its additional ZZ domain. Besides the multiple S/T residues of p62-D6, the D5 mutant contains an additional 10 Ser and Thr residues. Therefore, we generated 11 point mutants of the p62-D5 mutant, which involved the mutations of S266/T269/S272A along with a single mutation at each of the new sites with Ala. Subsequently, we further examined the effect of these mutations on DYRK3-mediated phosphorylation. The Fig. S1*B* illustrates the results of the *in vitro* kinase assay, which demonstrated that the p62-D5-S207/S266/T269/S272A mutant exhibited the most significant reduction in phosphorylation level among the 11 mutants. These findings indicate that DYRK3 can phosphorylate the site(s) of S207, S266, T269, or/and S272.

Next, we generated three p62 mutants with single substitutions at S266A, T269A, or S272A and examined the effect of these mutations on DYRK3-mediated phosphorylation. The *in vitro* kinase assay revealed that the S266A and S272A mutants were still significantly phosphorylated by DYRK3 (Fig. S1*C*). We then investigated whether S207 and/or T269 could be phosphorylated by DYRK3. To achieve this, we generated three additional p62 mutants, with single or double mutations at S207A and T269A, respectively. *In vitro* kinase assays of these mutants, followed by autoradiographic analyses, demonstrated that p62-S207/T269A exhibited the most substantial reduction in phosphosignal compared to p62-WT (Fig. 3, *D* and *E*). Furthermore, the p62-T269A mutant showed a significantly lower phosphorylation signal compared to p62-T207A, which was approximately 40% less than that of WT p62 (Fig. 3*E*).

Finally, we investigated whether this occurs within the cell. Following transfection of HEK293 cells with Myc-p62-WT, Myc-p62-S207/T269A, and/or Flag-DYRK3, cell lysates were immunoprecipitated using an anti-Myc antibody. Immunoblotting of the samples with anti-pT269/pS272-p62 or anti-pS207-p62 antibodies revealed a significant increase of DYRK3-mediated phosphorylation in p62-WT, as expected. However, this effect was not observed in the p62-S207/T269A mutant (Fig. 3, *F* and *G*).

We then investigated the colocalization of DYRK3 and phospho-p62 at T269/S272 in SK-Mel-28 melanoma cells. Immunostaining of the cells using anti-pT269/S272-p62 (red) or anti-DYRK3 (green) antibodies revealed that both endogenous proteins mainly colocalize within the cytoplasm, with a minor presence in the nucleus (Fig. S2). These findings were consistent with those observed in cells transiently transfected with DYRK3 and p62. Furthermore, these results suggest that DYRK3 might phosphorylate p62 at T269 both in the cytosol and the nucleus.

Taken together, these findings suggest that DYRK3 phosphorylates p62 at T269 and S207 within the TB domain, with a greater preference for T269. Since DYRK3 prominently catalyzes the phosphorylation of p62 at T269, we further assessed the impact of T269 phosphorylation on p62 function and its physiological consequences in the subsequent study.

### DYRK3 enhances the binding of p62 to TRAF6

**Figure 4:**
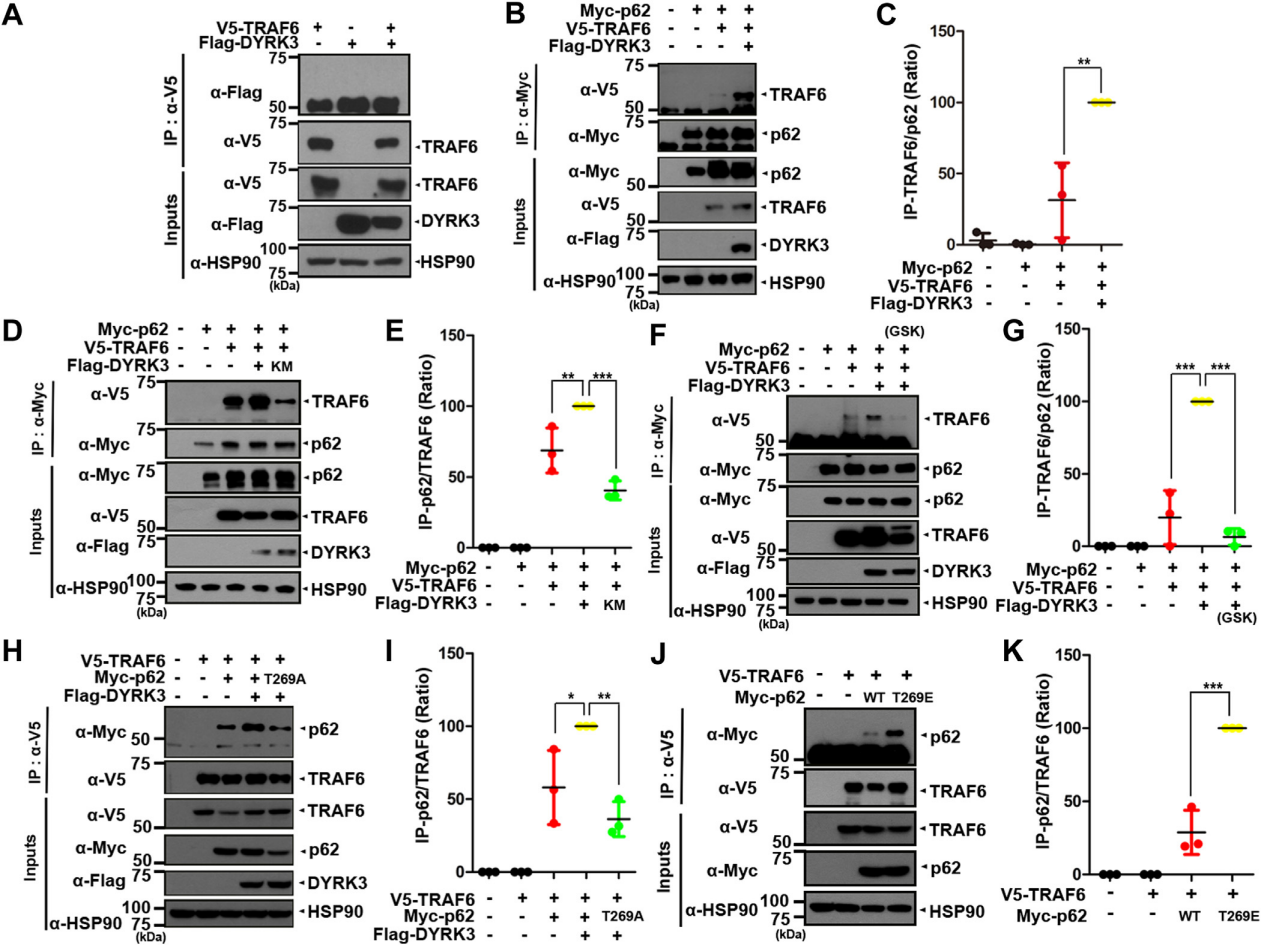
**DYRK3-mediated phosphorylation of p62 enhances its binding to TRAF6.***A*, after HEK293 cells were transfected for 24 h with a plasmid encoding V5-TRAF6 or/and Flag-DYRK3, cell lysates were immunoprecipitated with an anti-V5 antibody, followed by immunoblotting with the indicated antibody. Hsp90 served as a loading control. *B*, HEK293 cells were transfected for 24 h with a plasmid encoding Myc-p62, V5-TRAF6, or Flag-DYRK3 alone or in combination. Cell lysates were immunoprecipitated with an anti-Myc antibody, followed by immunoblotting with the indicated antibody. *C*, quantification of the protein band densities in (*B*) was performed. All data represent the mean ± standard deviation of three independent experiments (∗∗*p* < 0.01). *D*, where specified, HEK293 cells were transfected for 24 h with a plasmid encoding Myc-p62, V5-TRAF6, Flag-DYRK3-WT, or Flag- DYRK3-KM alone or in combination. Cell lysates were immunoprecipitated with an anti-Myc antibody, followed by immunoblotting with the indicated antibody. *E*, quantification of the band densities in (*D*) was performed. All data represent the mean ± standard deviation of three independent experiments (∗∗∗*p* < 0.001, ∗∗*p* < 0.01). *F*, after HEK293 cells were transfected for 24 h with a plasmid encoding Myc-p62, V5-TRAF6, or Flag-DYRK3-WT alone or in combination, the cells were then left untreated or treated for 6 h with 1 μM of GSK-626616. Cell lysates were immunoprecipitated with anti-Myc antibody, followed by immunoblotting with the indicated antibody. *G*, quantification of the band densities was performed in (*F*). All data represent the mean ± standard deviation of three independent experiments (∗∗∗*p* < 0.001). *H*, after HEK293 cells were transfected for 24 h with a plasmid encoding Myc-p62-WT, Myc-p62-T269A, V5-TRAF6, or Flag-DYRK3-WT alone or in combination, cell lysates were immunoprecipitated with an anti-V5 antibody, followed by immunoblotting with the indicated antibody. *I*, quantification of the protein blots in (*H*) was performed. All data represent the mean ± standard deviation of three independent experiments (∗∗*p* < 0.01, ∗*p* < 0.05). *J*, after HEK293 cells were transfected for 24 h with a plasmid encoding Myc-p62-WT, Myc-p62-T269E, or V5-TRAF6 alone or in combination, cell lysates were immunoprecipitated with an anti-V5 antibody, followed by immunoblotting with the indicated antibody. *K*, quantification of the band densities in (*J*) was performed. All data represent the mean ± standard deviation of three independent experiments (∗∗∗*p* < 0.001). DYRK, dual-specificity tyrosine-phosphorylation-regulated kinase; HEK, human embryonic kidney cells; TRAF6, tumor necrosis factor receptor–associated factor 6.

In a previous report, it was demonstrated that the TB domain of p62 interacts with TRAF6, thereby enhancing the ubiquitin E3 ligase activity of TRAF6 (19). Based on this finding, we investigated whether DYRK3-mediated phosphorylation affects the downstream interaction between p62 and TRAF6. As a control, we initially examined whether DYRK3 directly binds to TRAF6. The co-IP assay revealed no binding between DYRK3 and TRAF6 (Fig. 4*A*), indicating the absence of a biochemical interaction between DYRK3 and TRAF6.

We then investigated whether DYRK3 affects the interaction between p62 and TRAF6. Following transfection of cells with Myc-p62, V5-TRAF6, or Flag-DYRK3 alone or in combination, cell lysates were immunoprecipitated using an anti-Myc antibody. Immunoblotting of the samples with an anti-V5 antibody revealed that p62 binds to TRAF6 in the basal state, as expected. In addition, the interaction between p62 and TRAF6 was significantly enhanced in the presence of WT DYRK3 (Fig. 4, *B* and *C*), but not in the presence of DYRK3-KM (Fig. 4, *D* and *E*). Moreover, treatment of cells with GSK626616, a specific chemical inhibitor of DYRK3, resulted in a considerable reduction in the binding between p62 and TRAF6 (Fig. 4, *F* and *G*). These findings indicate that the kinase activity of DYRK3 positively contributes to the binding affinity between p62 and TRAF6.

We then investigated whether the phosphorylation of p62 at T269 influences its binding affinity to TRAF6. After transfecting cells with plasmids encoding Myc-tagged WT p62 or its phosphorylation-defective mutant (Myc-p62-T269A), V5-TRAF6, or Flag-DYRK3-WT alone or in combination (Fig. 4, *H* and *I*), cell lysates were immunoprecipitated with an anti-V5 antibody. Immunoblotting of the samples with an anti-Myc antibody revealed that the p62-T269A mutant exhibited substantially reduced binding to TRAF6 than WT p62. Similarly, the p62-T269E mutant displayed stronger binding to TRAF6 than WT p62 in the absence of DYRK3 (Fig. 4, *J* and *K*). Taken together, these results suggest that the phosphorylation of p62 at T269 by DYRK3 enhances its binding affinity with TRAF6.

### DYRK3 phosphorylates p62 to promote polyubiquitination of mTOR via K63-linkage

**Figure 5:**
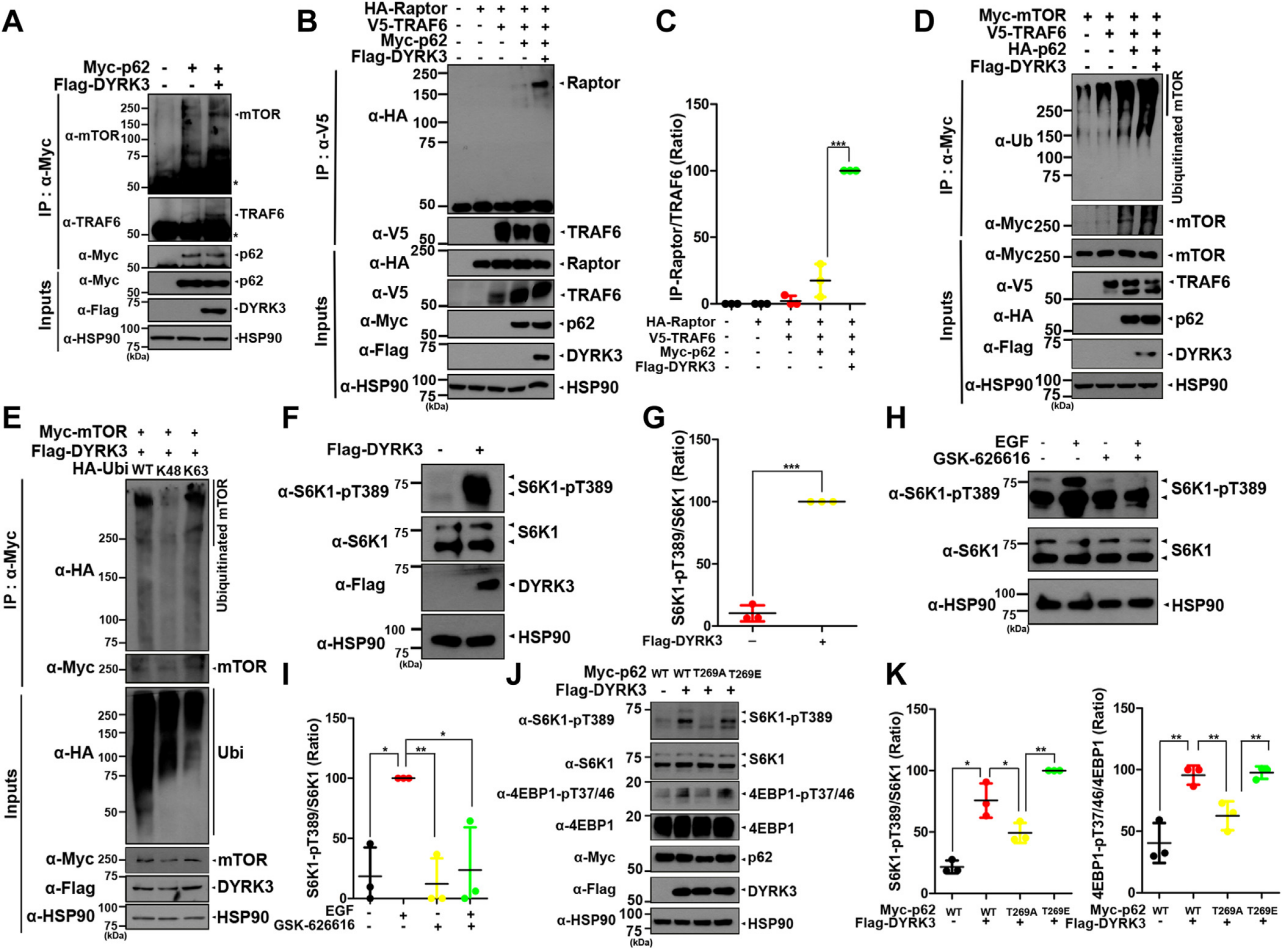
**Upon p62 phosphorylation, TRAF6 promotes the polyubiquitination of mTOR via K63-linkage.***A*, after HEK293 cells were transfected for 24 h with a plasmid encoding Myc-p62 or/and Flag-DYRK3, cell lysates were immunoprecipitated with an anti-Myc antibody, followed by immunoblotting with the indicated antibody. *B*, after HEK293 cells were transfected for 24 h with a plasmid encoding HA-Raptor, Myc-p62, V5-TRAF6, or Flag-DYRK3-WT alone or in combination, cell lysates were immunoprecipitated with an anti-V5 antibody, followed by immunoblotting with the indicated antibody. *C*, quantification of the protein blots in (*B*) was performed. All data represent the mean ± standard deviation of three independent experiments (∗∗∗*p* < 0.001). *D*, after HEK293 cells were transfected for 24 h with a plasmid encoding Myc-mTOR, V5-TRAF6, HA-p62, or Flag-DYRK3-WT alone or in combination, cell lysates were immunoprecipitated with an anti-Myc antibody, followed by immunoblotting with the indicated antibody. *E*, after HEK293 cells were transfected for 24 h with a plasmid encoding Myc-mTOR, HA-Ub-WT, HA-Ub-K48, HA-Ub-K63, or Flag-DYRK3-WT alone or in combination, cell lysates were immunoprecipitated with an anti-Myc antibody, followed by immunoblotting with the indicated antibody. *F*, after HEK293 cells were transfected for 24 h with a plasmid encoding Flag-DYRK3-WT, followed by immunoblotting with the indicated antibody. *G*, quantification of the protein blots in (*F*) was performed. All data represent the mean ± standard deviation of three independent experiments (∗∗∗*p* < 0.001). *H*, where indicated, cells were left untreated or treated for 6 h with 1 μM GSK-626616 or/and 100 ng/ml EGF, followed by immunoblotting with the indicated antibody. *I*, quantification of the protein blots in (*H*) was performed. All data represent the mean ± standard deviation of three independent experiments (∗∗*p* < 0.01, ∗*p* < 0.05). *J*, after HEK293 cells were transfected for 24 h with a plasmid encoding Myc-p62-WT, Myc-p62-T269A, Myc-p62-pT269E, or Flag-DYRK3-WT alone or in combination, followed by immunoblotting with the indicated antibody. *K*, quantification of the blots in (*J*) was performed. All data represent the mean ± standard deviation of three independent experiments (∗∗*p* < 0.01, ∗*p* < 0.05). DYRK, dual-specificity tyrosine-phosphorylation-regulated kinase; EGF, epidermal growth factor; HA, hemagglutinin; HEK, human embryonic kidney cells; TRAF6, tumor necrosis factor receptor–associated factor 6.

Upon the binding of TRAF6 to p62, TRAF6 facilitates the ubiquitination of mTOR, leading to the activation of the mTORC1 pathway (20). Based on this finding, we further investigated whether the phosphorylation of p62 at T269 by DYRK3 influences the mTORC1 pathway. Following transfection of cells with Myc-p62 or Flag-DYRK3 alone or in combination, cell lysates were immunoprecipitated using an anti-Myc antibody. Immunoblotting of the samples with anti-mTOR and anti-TRAF6 antibodies revealed that p62 binds to both mTOR and TRAF6 in the basal state, as expected. Moreover, the formation of the complex between p62, TRAF6, and mTOR was significantly enhanced in the presence of WT DYRK3 (Fig. 5*A*).

In the mTORC1 pathway the mTORC1 complex specifically contains Raptor as a scaffolding protein, which links the mTOR kinase with mTORC1 substrates and facilitates mTORC1 signaling (21). We then examined whether the phosphorylation of p62 at T269 might influence the binding affinity of TRAF6 to Raptor within the mTORC1 complex. After transfecting cells with plasmids encoding Myc-tagged p62, hemagglutinin (HA)-Raptor, V5-TRAF6, or Flag-DYRK3-WT alone or in combination (Fig. 5, *B* and *C*), cell lysates were immunoprecipitated with an anti-V5 antibody. Immunoblotting of the samples with an anti-HA antibody revealed that the phosphorylation of p62 at T269 by DYRK3 significantly increased the binding between TRAF6 and Raptor. These results suggest that the phosphorylation of p62 at T269 by DYRK3 enhances the binding affinity of p62 with mTORC1.

We further investigated whether the phosphorylation of p62 at T269 affects the E3 ligase activity of TRAF6 toward mTOR. To address this, cells were transfected with plasmids encoding Myc-mTOR, V5-TRAF6, HA-p62, or Flag-DYRK3-WT alone or in combination. The co-IP of cell lysates using anti-ubiquitin, followed by immunoblotting with anti-Myc antibody, revealed that the presence of p62 enhances the polyubiquitination of mTOR in cells cotransfected with DYRK3 and TRAF6 (Fig. 5*D*). Additionally, the expression of the HA-ubiquitin-K48 mutant, in which all lysine residues of ubiquitin except K48 were replaced with arginine, impaired the polyubiquitination of mTOR, whereas the HA-ubiquitin-K63 mutant, where all lysine residues of ubiquitin except K63 were replaced with arginine, showed no effect (Fig. 5*E*). These results demonstrate that the polyubiquitination of mTOR induced by DYRK3-mediated p62 phosphorylation occurs through K63-linkage, rather than K48. K48-linked ubiquitination typically targets proteins for degradation, while K63-linked ubiquitination often regulates the biochemical and functional properties of targets, such as intracellular localization, catalytic activity, or downstream signaling cascade (22).

Lastly, we examined whether and how the kinase activity of polyubiquitinated mTOR is affected after DYRK3-mediated phosphorylation of p62 at T269. In several studies, S6K and 4EBP1 have commonly been used as substrates of mTOR kinase (23, 24). To investigate this, cells were transfected with a plasmid encoding Flag-DYRK3, followed by immunoblotting of cell lysates with anti-pT389-S6K antibody. As shown in Figure 5, *F* and *G*, overexpression of DYRK3 increased the phosphorylation of S6K, compared to mock-transfected cells. Various stimuli, including epidermal growth factor (EGF) and insulin, can promote the activation of mTORC1 in mammalian cells (25). Consistent with this finding, we also observed that stimulation of cells with EGF resulted in the phosphorylation of S6K at T389. Additionally, treatment of cells with the specific inhibitor of DYRK3 (GSK-626616) reduced the phosphorylation of S6K at T389 in EGF- and insulin-stimulated HEK cells, indicating impaired mTORC1 activity (Fig. 5, *H* and *I*). Furthermore, the phosphorylation of p62 at T269 by DYRK3 increased the phosphorylation of S6K at T389, as well as the phosphorylation of 4EBP1 at T37/46 (Fig. 5, *J* and *K*). After cells were transfected with Myc-p62-WT, Myc-p62-T269A, Myc-p62-T269E, or Flag-DYRK3 alone or in combination, immunoblotting of the samples with anti-pT269-S6K and anti-pT37/46-4EBP1 antibodies revealed that the phosphorylation of S6K and 4EBP1 was reduced in the presence of p62-T269A compared to cells with p62-WT or p62-T269E. These data suggest that the phosphorylation of p62 at T269 promotes the activation of mTORC1 and its downstream signaling pathway.

### The sequential activation of the DYRK3-p62-TRAF6-mTORC1 pathway is relevant to the growth of melanoma cancer cells

**Figure 6:**
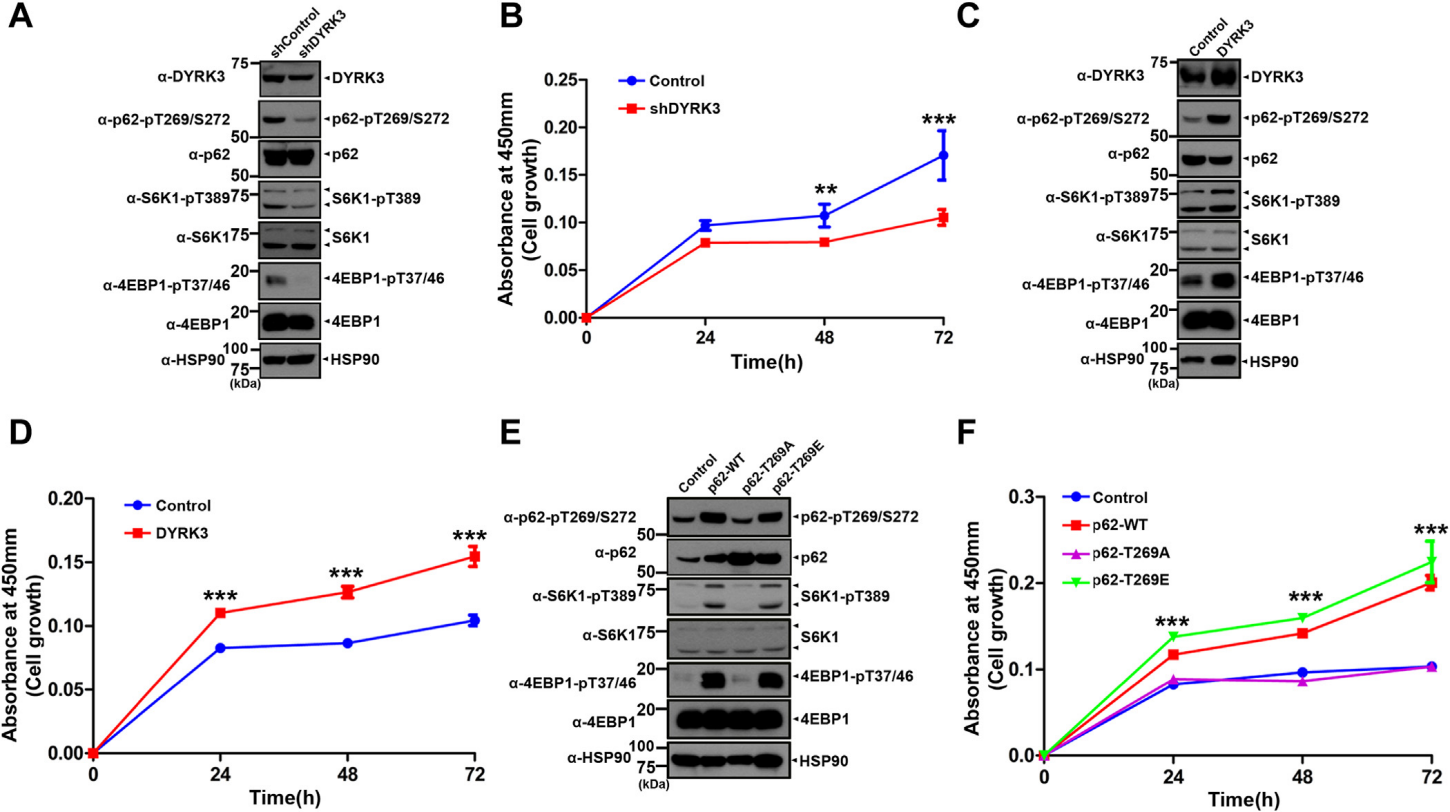
**The sequential activation of the DYRK3-p62-TRAF6-mTORC1 pathway stimulates the growth of melanoma cancer cells.***A*, after SK-Mel-28 cells were transfected for 24 h with a plasmid encoding scrambled control shRNA or shRNA-*DYRK3*, immunoblotting was performed with the indicated antibody. *B*, SK-Mel-28 cell lines were transfected with either control shRNA or shRNA-*DYRK3,* and cell viability was counted using the CCK-8 assay. Data are represented as the mean ± standard deviation of six independent experiments (∗∗∗*p* < 0.001, ∗∗*p* < 0.01). *C*, cell lysates were prepared from SK-Mel-28 cells stably expressing mock vector (Mock) or DYRK3 and subjected to Western blotting with the indicated antibody. *D*, cell viability of SK-Mel-28 cell lines stably expressing mock vector (control) or DYRK3 was measured using the CCK-8 assay. Data are represented as the mean ± standard deviation of six independent experiments (∗∗∗*p* < 0.001). *E*, cell lysates were prepared from SK-Mel-28 cells stably expressing mock vector (control), p62-WT, p62-T269A, or p62-T269E and subjected to Western blotting with the indicated antibody. *F*, cell viability of SK-Mel-28 cell lines stably expressing mock vector (control), p62-WT, p62-T269A, or p62-T269E was measured using the CCK-8 assay. Data are represented as the mean ± standard deviation of six independent experiments (∗∗∗*p* < 0.001). CCK-8, cell counting kit-8; DYRK, dual-specificity tyrosine-phosphorylation-regulated kinase; TRAF6, tumor necrosis factor receptor–associated factor 6.

Based on the findings that the upregulation of DYRK3 in melanoma skin cancer cells and dysregulation of the mTOR pathway are closely associated with various types of human tumors, we investigated the effect of DYRK3-mediated activation of the mTORC1 pathway on melanoma cancer cell growth. To explore this aspect, human SK-Mel-28 melanoma cancer cells were transfected with a plasmid encoding *DYRK3-*shRNA. Immunoblot of cell lysates with anti-pT269/S272-p62, anti-pT389-S6K, or anti-pT37/46-4EBP1 antibodies revealed that *DYRK3* knockdown decreases the phosphorylation of p62, S6K, and 4EBP1 compared to cells transfected with scrambled shRNA as a control (Fig. 6*A*). Moreover, *DYRK3* knockdown decreased the growth of melanoma cancer cells, compared to control cells (Fig. 6*B*). Furthermore, when we established human SK-Mel-28 melanoma cells stably expressing DYRK3, they exhibited much enhanced phosphorylation of p62, S6K, and 4EBP1 compared to mock-transfected control cells (Fig. 6*C*). Additionally, overexpression of DYRK3 resulted in increased melanoma cancer cell growth compared to control cells (Fig. 6*D*). We then examined whether the phosphorylation of p62 at Thr-269 influences the growth of melanoma cancer cells. For this analysis, we established SK-Mel-28 melanoma cells stably expressing p62-WT, p62-T269A, or p62-T269E (Fig. 6*E*). Immunoblotting of the samples with anti-pT269-S6K and anti-pT37/46-4EBP1 antibodies revealed that the phosphorylation of S6K and 4EBP1 is reduced in cells with p62-T269A compared to cells with p62-WT or p62-T269E. Furthermore, the melanoma cancer cell growth was significantly decreased by transfection of p62-T269A, but not by p62-WT or p62-T269E (Fig. 6*F*). In addition, immunoblotting of the samples with anti-pT269-S6K and anti-pT37/46-4EBP1 antibodies revealed that the phosphorylation of S6K and 4EBP1 is reduced in cells with p62-T269A compared to cells with p62-WT in SK-Mel-28-DYRK3 stable melanoma cell (Fig. S3, *A* and *B*). These results suggest that DYRK3 may not directly phosphorylate S6K and 4EBP1, but rather, the phosphorylation of these proteins occurs through p62-T269 phosphorylation.

Taken together, these results indicate that the phosphorylation of p62 at T269 by DYRK3 promotes the growth of human SK-Mel-28 melanoma cancer cells.

### The activation of the DYRK3-p62-TRAF6-mTORC1 pathway promotes melanoma cancer progression

**Figure 7:**
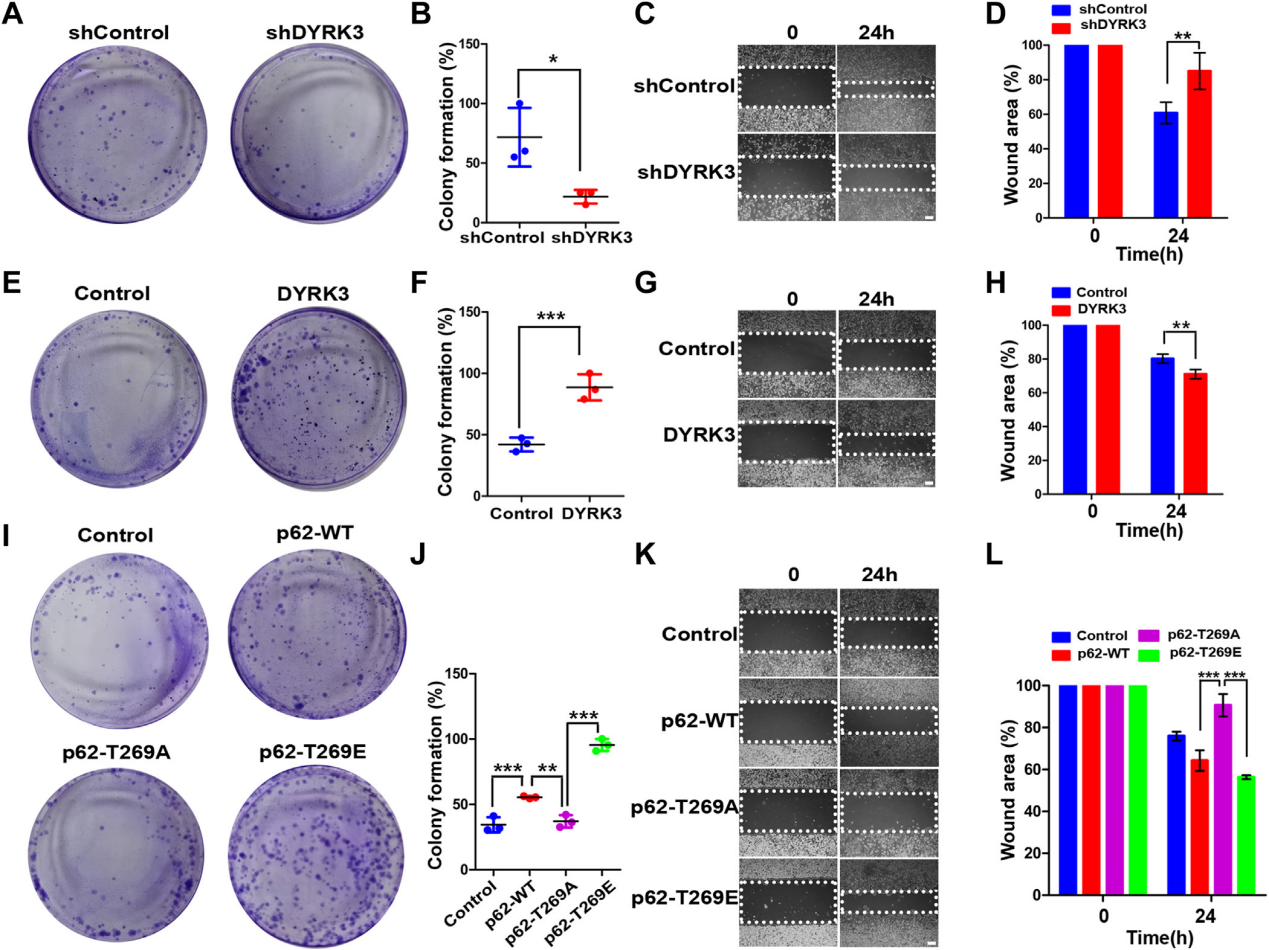
**The activation of the DYRK3-p62-TRAF6-mTORC1 pathway enhances the progression of melanoma skin cancer.***A* and *B*, SK-Mel-28 cell lines transfected with control shRNA or shRNA-*DYRK3* were cultured for 10 days, and stained with crystal violet, and their colony formation percentages were determined. Data are presented as the mean ± standard deviation of three independent experiments. *C* and *D*, SK-Mel-28 cells were transfected with control shRNA or shRNA-*DYRK3* for 24 h, plated for an additional 24 h, and then subjected to a wound-healing assay by scratching the monolayers with a pipette tip. The cell migration rate was assessed after 18 h of incubation and visualized under a microscope. Data are presented as the mean ± standard deviation of three independent experiments (∗∗*p* < 0.01). The scale bar represents 10 μm. *E* and *F*, SK-Mel-28 cells stably expressing mock vector (control) or DYRK3 were cultured for 10 days, and stained with crystal violet, and their colony formation percentages were determined. Data are presented as the mean ± standard deviation of three independent experiments (∗∗∗*p* < 0.001). *G* and *H*, SK-Mel-28 cell lines stably expressing mock vector (control) or DYRK3 were plated for 24, subjected to a wound-healing assay by scratching the monolayers with a pipette tip, and incubated for 18 h. The cell migration rate was assessed and viewed under a microscope. Data are presented as the mean ± standard deviation of three independent experiments (∗∗*p* < 0.01). The scale bar represents 10 μm. *I* and *J*, SK-Mel-28 cells stably expressing mock vector (control), p62-WT, p62-T269A, or p62-T269E were plated for 24 h, subjected to a wound-healing assay, and incubated for 18 h. The cell migration rate was assessed and viewed under a microscope. Data are presented as the mean ± standard deviation of three independent experiments (∗∗∗*p* < 0.001, ∗∗*p* < 0.01). *K* and *L*, SK-Mel-28 cells stably expressing mock vector (control), p62-WT, p62-T269A, or p62-T269E were plated for 24 h, subjected to a wound-healing assay, and incubated for 18 h. The cell migration rate was assessed and viewed under a microscope. Data are presented as the mean ± standard deviation of three independent experiments (∗∗∗*p* < 0.001). The scale bar represents 10 μm. DYRK, dual-specificity tyrosine-phosphorylation-regulated kinase; TRAF6, tumor necrosis factor receptor–associated factor 6.

Next, we assessed the effect of sequential activation of the DYRK3-p62-TRAF6-mTORC1 pathway on melanoma cancer progression. The colony formation assay showed that cells with *DYRK3* knockdown exhibited a decrease in colony numbers compared to control cells (Fig. 7, *A* and *B*), Moreover, wound-healing assays revealed that cells with *DYRK3* knockdown displayed a lower migration potential compared to cells with control shRNA (Fig. 7, *C* and *D*). Conversely, cells stably overexpressing DYRK3 exhibited a significant increase in the colony formation index (Fig. 7, *E* and *F*) and greatly enhanced migration potential compared to mock-transfected control cells (Fig. 7, *G* and *H*). We next examined whether the phosphorylation of p62 at Thr-269 influences melanoma cancer cell proliferation. The colony formation assay showed that cells transfected with p62-T269A exhibited a decrease in colony numbers compared to cells with p62-WT or p62-T269E (Fig. 7, *I* and *J*). Similarly, the wound-healing assay revealed that cells overexpressing p62-T269A displayed migration at a greatly reduced rate compared to cells with p62-WT or p62-T269E (Fig. 7, *K* and *L*).

Taken together, these results indicate that phosphorylation of p62 at T269 by DYRK3 promotes the progression of SK-Mel-28 human melanoma cancer cells.

### The activation of the DYRK3-p62-TRAF6-mTORC1 pathway promotes melanoma cancer cell growth *in vivo*

**Figure 8:**
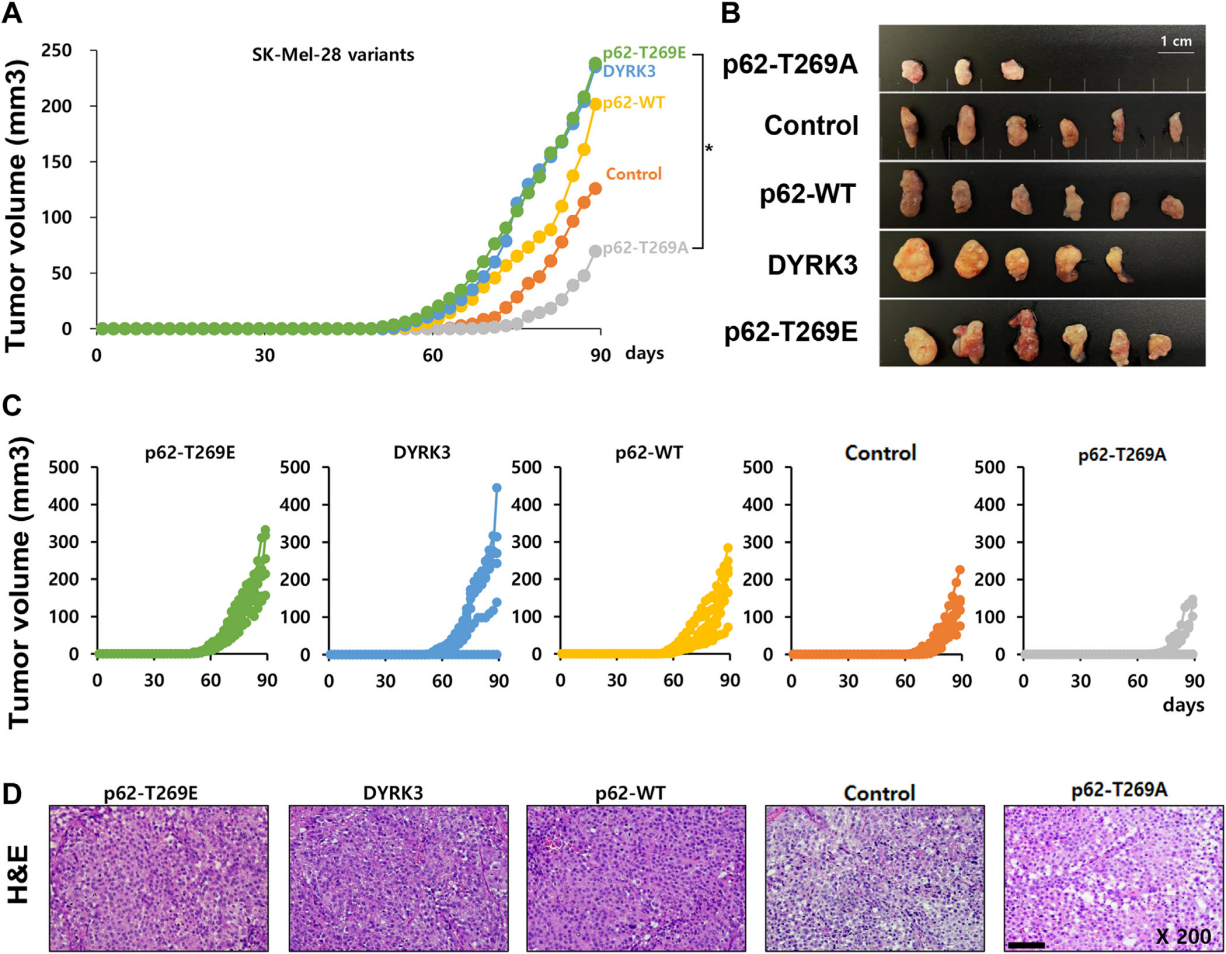
**The activation of the DYRK3-p62-TRAF6-mTORC1 pathway promotes the progression of melanoma skin cancer in nude mice.***A*, SK-Mel-28 cells expressing p62 variants were subcutaneously injected into mice. Tumor growth was monitored every 3 days until the end of the study. Values represent the mean ± S.E.M. for four animals per group (*p*∗ < 0.05). *B*, tumor tissues harvested from each group were visualized. *C*, individual growth curve of each mouse in each group was monitored. *D*, tumors were harvested on day 21 for histological analysis. H&E staining was performed on tumor sections from each group of mice. The scale bar represents 100 μm. DYRK, dual-specificity tyrosine-phosphorylation-regulated kinase; TRAF6, tumor necrosis factor receptor–associated factor 6.

To further investigate the effect of DYRK3-mediated p62 phosphorylation on *in vivo* tumorigenesis, several SK-Mel-28 cell lines stably expressing DYRK3-WT, p62-WT, p62-T269A, or p62-T269E, as well as mock-transfected control cells, were subcutaneously injected into nude mice, and tumor growth was monitored. As shown in Figure 8, *A*–*C*, SK-Mel-28 tumors expressing p62-WT reached 201.8 ± 28.1 mm^3^ by day 89 following subcutaneous inoculation of cancer cells. The growth of mock-transfected or p62-T269A-expressing SK-Mel-28 tumors was slower than that of p62-WT-expressing SK-Mel-28 tumors, with average tumor volumes reaching 126.0 ± 24.9 or 69.5 ± 26.5 mm^3^, respectively. Only 50% of the injected mice showed successful tumor engraftment in the p62-T269A group. Both SK-Mel-28 tumors expressing p62-T269E or DYRK3 reached an average volume of 235.4 ± 56.8 or 238.5 ± 28.6 mm^3^, respectively, showing about 1.9-fold or 3.4-fold higher tumor volume compared to mock-transfected SK-Mel-28 or p62-T269A-expressing SK-Mel-28 tumors. Throughout the course of the study, no systemic toxicity, such as diarrhea, weight loss, or cachexia, was observed. Histological analysis of the tumor tissues revealed that the necrotic region was scarce in SK-Mel-28 expressing p62-T269E or p62-WT, whereas tumors from mock-transfected SK-Mel-28 or p62-T269A-expressing SK-Mel-28 tumors presented with extensive necrosis (Fig. 8*D*). Taken together, these results suggest that phosphorylation of p62 at T269 by DYRK3 promotes SK-Mel-28 tumor growth *in vivo*.

## Discussion

Several kinases have been reported to directly phosphorylate p62 at multiple sites, including PKA, LRRK2, CDK1, MEKK3, MEK3/6, p38δ, AMPK, CK1, TAK1, CK2, TBK1, and ULK1. These kinases phosphorylate p62 at various sites, such as S24, T138, S176, T269, S272, S294, S349, S403, or S407. Specifically, phosphorylation of p62 at Ser-24 by PKA inhibits its oligomerization with other PB1-containing proteins (26). Additionally, autosomal-dominant missense mutations in the *LRRK2* gene are the most common genetic predisposition for developing Parkinson's disease. A recent study has identified p62 as a novel substrate of LRRK2 (27). Phosphorylation of T138 and S176 in the ZZ domain of p62 by LRRK2 promotes its binding affinity for ubiquitinated cargos destined for autophagic degradation in response to neuronal toxicity (27). Moreover, AMPK phosphorylates p62 at Ser-294 upon insulin withdrawal, inducing the mitochondrial translocation of p62 and mitophagy (28). Phosphorylation of p62 by AMPK on Ser-294 is also necessary for autophagic cell death in adult rat hippocampal neural cells.

Regarding the phosphorylation of p62 at S207, a study reported an increase in the amount of phosphorylated p62 at S207 in Neuro2a cells in response to the proteasome inhibition (29), but the kinase(s) responsible for this phosphorylation was not identified. In the present study, we identified for the first time that DYRK3 phosphorylates p62 at S207, although the functional or physiological consequences of this phosphorylation have not been further clarified. We also found that DYRK3 targets and phosphorylates T269, positively influencing the downstream TRAF6-mTORC1 pathway. As for other kinase(s) targeting to T269, MAPK13 (p38δ), and CDK1 have been identified to phosphorylate p62 at T269 and S272 to date, and modification of p62 at these two sites appears to be involved in either mTORC1-dependent autophagy inhibition or mitotic progression (30, 31). Specifically, CDK1 phosphorylates p62 at T269 and S272, which is necessary for maintaining appropriate cyclin B1 levels and the levels of CDK1 activity required for proper entry into and exit from mitosis. The absence of CDK1-mediated phosphorylation of p62 results in a faster exit from mitosis, leading to enhanced cell proliferation and tumorigenesis in response to Ras-induced transformation.

Moreover, phosphorylation of p62 at S349 in the Keap1-interacting region domain by CK1, TAK1, or PRKCD enhances its interaction with Keap1 to regulate the Nrf2 antioxidant system (32, 33, 34, 35). Both the Keap1-Nrf2 system and autophagy play roles in the oxidative-stress response, metabolic pathways, and innate immunity, and dysregulation of these processes is associated with pathological conditions (36). Lastly, ULK1, CK2, or TBK1 can phosphorylate p62 at S403 (37, 38, 39). The fact that p62 could be phosphorylated at multiple sites by various kinases indirectly suggests the functional and physiological importance of p62 in cellular homeostasis. Therefore, its activity should be precisely and efficiently regulated and maintained through multiple modulatory and backup mechanisms.

Here, we demonstrated that DYRK3 binds to p62 through the central region spanning the ZZ and TB domains and phosphorylates p62 at S207 and T269 within the TB domain. Considering the functional role of the TB domain in p62, cells recruit Rag proteins to the lysosome in response to amino acid stimulation (40). The Rag proteins then interact with a raptor, a key step for mTORC1 activation. Subsequently, p62 interacts with raptor to recruit mTORC1 to the lysosome, where it is activated by the mTORC1 activator Rheb (41). Additionally, p62 recruits TRAF6 through the TB domain, and TRAF6-mediated ubiquitination of mTORC1 leads to mTORC1 activation (12). The TB domain contains a TRAF6-binding motif (PSEDP, amino acids 228-232) that conforms to the canonical TRAF6 consensus binding site (PXEXX) (42, 43). To assess the importance of TRAF6 interaction with p62 for mTORC1 activation, deletion of the TB domain completely abolished the interaction of p62 with TRAF6. This indicates that the TB sequence is necessary for the p62-TRAF6 interaction, and residues 228, 230, and 232 are critical for binding (20). Therefore, phosphorylation in the TB domain of p62 could alter the protein structure to enhance its binding to TRAF6, as exemplified by our findings. As anticipated, our study provides additional evidence that DYRK3-mediated phosphorylation of p62 at T269 in the TB domain affects the interaction between TRAF6 and mTORC1, positively regulating the mTORC1 pathway.

Regarding the physiological association between DYRK3 and cancer, a recent study discovered that radiation-induced expression of DYRK3 leads to increased mitochondrial fission through mTORC1-dependent DRP1 activation, thereby promoting brain tumors like glioblastoma multiforme (GBM) (3). Their analysis of the The Cancer Genome Atlas database revealed a significant elevation in *DYRK3* mRNA levels in GBM patients compared to normal controls. Following irradiation, DYRK3 levels were increased, enhancing mTORC1 activity through PRAS40 phosphorylation and activating DRP1, a regulator of mitochondrial fission. Furthermore, it was observed that ERK1/2 phosphorylates DRP1 at Ser-616, promoting RAS-induced melanoma cell growth (44). These studies further support the present finding of a strong association between DYRK3 and the promotion of melanoma cancer. Moreover, our results suggest that DYRK3-mediated phosphorylation of p62 could contribute to the development of GBM, and it would be intriguing to validate this hypothesis through further studies.

Melanoma cells acquire the ability for uncontrolled proliferation, evasion of cellular senescence, resistance to apoptosis, and metastatic spread during disease progression. It is likely that a combination of factors, including environmental and genetic factors, contributes to the development of melanoma. There is growing recognition for the impact of DNA methylation alterations in conferring these malignant characteristics to early-stage tumor cells (45). Therefore, the identification of epigenetic changes in melanoma could be applied to molecular diagnosis and prognosis of the disease. Additionally, approximately 10% of melanomas are caused by gene mutations, with BRAF being an example. BRAF is a serine/threonine-specific protein kinase that is mutated in 50∼70% of cutaneous melanomas. The most common mutation in BRAF in melanoma (found in over 90% of cases) is a substitution of valine with glutamic acid at position 600 (V600E) (46). The BRAF mutation V600E also stimulates melanoma cell invasion *in vitro* and plays a role in tumor neoangiogenesis *in vivo* (47). Several other genes, including CDKN2A, CDK4, IDH1, EZH2, PPP6C, RAC1, SNX31, TACC1, STK19, and ARID2, have also been primarily associated with familial melanoma. In addition to these genes, the present study proposes two additional markers for melanoma progression: p62 and DYRK3. Interestingly, the expression levels of p38δ are significantly elevated in primary tumors of various cancers, including skin cancer (48, 49). Similar to the action of DYRK3, p38δ can phosphorylate p62 at T269 and S272, and p38δ is known to promote skin carcinogenesis by facilitating the formation of a proinflammatory microenvironment that supports epidermal hyperproliferation and tumorigenesis (50). Based on our findings, the melanoma-promoting activity of p38δ could be mediated through the activation of the p62-TRAF6-mTROC1 signaling pathway.

In the present study, we utilized several melanoma cell lines, including SK-Mel-28, SK-Mel-5, UACC62, UACC257, M14, LOX-IMVI, and SK-Mel-2. BRAF-V600E mutations were observed in almost all melanoma cell lines except for the SK-Mel-2 cell line. A previous report found that DYRK1A enhances neural growth factor-induced differentiation of PC12 cells through its interaction with components of the Ras-BRaf-MEK1 signaling cascade (51). Additionally, the expression level of p62 was upregulated in melanoma cells compared to normal human epidermal melanocytes, and UV irradiation also upregulated p62 expression in epidermal melanocytes and melanoma cells (52). Supporting this observation, *p62*-KO mice with overexpression of BRAF-V600E showed inhibition of melanoma development and metastasis (53). The present study further suggests that high expression of p62 and DYRK3 in melanoma can promote cancer growth via the mTOR pathway. Since all the melanoma cell lines we used exhibited higher levels of p62 and DYRK3, this characteristic appears to be independent of the BRAF-V600E mutation.

In conclusion, we propose a novel oncogenic mechanism of DYRK3 in melanoma cancer cells, involving the sequential steps of p62 phosphorylation at T269 and subsequent activation of the TRAF6-mTORC1-mTOR pathway.

## Experimental procedures

### Materials

Dulbecco's modified Eagle medium (DMEM), fetal bovine serum (FBS), and ProLong Gold Antifade Mountant with 4′,6-diamidino-2-phenylindole were purchased from Invitrogen. Protein A-Sepharose and Ni-NTA agarose beads were purchased from GE HealthCare Life Sciences. Enhanced chemiluminescence reagents were purchased from AbClon. RPMI-1640 medium and PEI were purchased from Sigma-Aldrich.. The following antibodies (and their catalog numbers) were purchased from the indicated vendors: Alexa Fluor 488–conjugated mouse IgG (A-11029) and Alexa Fluor 594–conjugated rabbit IgG (A-11012), and anti-V5 (R960-25) antibodies from Invitrogen, and mouse monoclonal anti-Flag antibody (F3165-1MG) and rabbit polyclonal anti-Flag (F7425-2MG) antibody from Sigma-Aldrich. Horseradish peroxidase–conjugated anti-rabbit (AP132P) and anti-mouse (AP124P) secondary antibodies were purchased from EMD Millipore. Mouse anti-p62 (ab56416) and polyclonal anti-V5 antibodies (ab9116) were purchased from Abcam. Mouse monoclonal anti-DYRK3 (sc-390532), anti-Hsp90 (sc-13119), mouse anti-GFP (sc-9996), mouse anti-TRAF6 (sc-7221), anti-ubiquitin (sc-8017), mouse anti-HA (sc-7392) and anti-tubulin (sc-8017) antibodies were purchased from Santa Cruz Biotechnology. Polyclonal anti-HA (PAB0861) and anti-Myc (PAB10345) antibodies were obtained from Abnova. Rabbit anti-phospho-Thr269/Ser272-p62 (#13121S), anti-mTOR (#2972), anti-S6K (#9202), anti-phospho-Thr389-S6K (#9205), anti-4E-BP1 (#9452), and anti-phospho-Thr37/46-4E-BP1 (#2855) antibodies were purchased from Cell Signaling Technology. Rabbit phospho-S207-p62 antibodies (OAAB16350) were purchased from Aviva Systems Biology.

### DNA constructs

Mammalian constructs encoding human WT DYRK3 with an N-terminal Flag tag (pRK5-Flag-DYRK3-WT) and its kinase-inactive mutant with the substitution of K238M (pRK5-Flag-DYRK3-KM) were prepared using the QuikChange XL site-directed mutagenesis kit (Agilent Technologies). Plasmids encoding either Myc-tagged WT p62 (pcDNA3.1-Myc-p62) or its deletion mutant (pcDNA3.1-Myc-p62-D1∼-D7) were kindly provided by Y. T. Kwon (Seoul National University School of Medicine, Seoul, Korea). Plasmids encoding HA-tagged WT Raptor (pcDNA3.1-HA-Raptor), Myc-tagged mTOR (pRK5-Myc-mTOR), and HA-tagged WT p62 (pCl-neo-HA-p62) were kindly provided by H. W. Park (Yonsei University, Seoul, Korea), S.H. Um (Sungkyunkwan University), and Y. J. Oh (Yonsei University), respectively. The lentiviral vectors including pLV-shRNA-EGFP-puro-hDYRK3 (VB900069-9664vgt), pLV-Exp-EGFP-puro-hDYRK3 (VB900007-7142uxs), and hp62 (VB900110-4174vda) were purchased from VectorBuilder Inc. To generate constructs encoding various p62 point-mutants with single or double amino acid substitutions, such as p62-T269A and T269E, site-directed mutagenesis reactions were performed using the QuikChange XL site-directed mutagenesis kit. All complementary DNA constructs were confirmed by DNA sequencing (BIONICS).

### Cell culture and DNA transfection

Human dopaminergic neuroblastoma SH-SY5Y cells, human epithelioid cervix carcinoma HeLa cells, human embryonic kidney 293 (HEK293 and HEK293T), and human melanoma cells including M14, SK-Mel-28, SK-Mel-2, and SK-Mel-5 cells were maintained in DMEM containing 10% FBS and 100 U/ml penicillin-streptomycin. Human prostatic epithelial RWPE-1 cells and human melanoma cells including Lox-IMVI, UACC257, and UACC62 cells were maintained in RPMI 1640 containing 10% FBS and 100 U/ml penicillin-streptomycin. Cells were grown at 37 °C in a 5% CO_2_ incubator. All DNA transfections were performed using PEI, Lipofectamine 2000, and Lipofectamine Plus reagents (Invitrogen), according to the manufacturer’s protocol. The RWPE-1 cells were kindly provided by M. Y. Kim (Yonsei University). The melanoma cell line including LOX-IMVI, UACC-62, UACC-257, M14, and SK-MEL-28 were kindly provided by J. H. Lee (University of Ulsan College of Medicine, Seoul, Korea). The melanoma cell lines including SK-MEL-2 and SK-MEL-5 were kindly provided by J. S. Kim (Sookmyung Women’s University, Seoul, Korea).

### Co-immunoprecipitation and Western blot analysis

Cell lysates were prepared by rinsing cells with ice-cold PBS and lysed with 1% Nonidet P-40 lysis buffer (50 mM Tris, pH 7.5, 150 mM NaCl, 1% Nonidet P-40, 10% glycerol, 0.2 mM PMSF, 1 mM Na_3_VO_4_, 10 mM NaF, and 1× protease inhibitor cocktail including 1 μg/ml aprotinin, 1 μg/ml leupeptin, and 1 μg/ml pepstatin). The cells were then scraped, and supernatants were collected after centrifugation at 15,700*g* for 15 min at 4 °C. For immunoprecipitation, 1 μg of the appropriate antibody was incubated with 1000 μg of cell lysate overnight at 4 °C with gentle rotation. The mixture was then incubated with 30 μl of a 1:1 mixture of protein A-Sepharose bead suspension for 2 h at 4 °C. The beads were pelleted by centrifugation at 9300*g* for 1 min and washed three times with 1% Nonidet P-40 lysis buffer. The immunocomplexes were boiled in SDS-PAGE sample buffer, separated by SDS-PAGE, and transferred to nitrocellulose membranes (Millipore). The membranes were blocked for 1 h at room temperature using Tris-buffered saline with Tween ([TBST]; 25 mM Tris, pH 7.5, 150 mM NaCl, and 0.1% Tween20) containing 5% nonfat dry milk and then incubated overnight at 4 °C in TBST containing the appropriate antibody. The membranes were washed three times in TBST and incubated with the horseradish peroxidase-conjugated secondary antibody for 2 h. The blots were then washed three times with TBST for 10 min, and the protein bands were visualized using enhanced chemiluminescence reagents (AbClon), according to the manufacturer’s instructions.

### *In vitro* kinase assay of DYRK3

After transfecting HEK293 cells with plasmids encoding Myc-tagged WT p62 or one of its point mutants for 24 h, cells were lysed in 1% NP-40 lysis buffer. Cell lysates were immunoprecipitated overnight at 4 °C with anti-Myc antibodies, and the resulting anti-Myc immunocomplexes were used as the substrate source of p62. For the kinase assay, the immunocomplexes containing Myc-tagged p62 were mixed with 1 μg of bacterial recombinant DYRK3 protein and 1× reaction buffer, which included 0.2 mM Na_3_VO_4_ and 10 μM ATP. The *in vitro* kinase reaction was initiated by adding 10 μCi [γ-^32^P]ATP, allowed to proceed for 30 min at 30 °C, and terminated by the addition of SDS-PAGE sample buffer. The samples were separated by SDS-PAGE, and the incorporated [γ-^32^P]ATP radioisotope was detected by autoradiography. Bacterial recombinant DYRK3 protein was generously provided by H. S. Cho (Yonsei University).

### Confocal microscopic analysis

HEK293 and SK-Mel-28 cells were seeded onto poly-L-lysine-coated coverslips in 6-well plates and allowed to reach approximately 50 to 70% confluence at 37 °C. After 24 h of DNA transfection, cells were washed twice with PBS (pH 7.4), fixed with 7.3% formaldehyde for 30 min, permeabilized with 0.2% Triton X-100 for 30 min, and blocked with 1% bovine serum albumin for 30 min at room temperature. Subsequently, the cells were stained with anti-Flag or anti-Myc antibodies, and fluorescein isothiocyanate-conjugated secondary antibodies were used to detect the primary antibodies. The samples were counterstained with 4′,6-diamidino-2-phenylindole, mounted, and analyzed using an LSM 980 confocal microscopy (Carl Zeiss). The acquired data were processed using Zeiss LSM Image Browser (Carl Zeiss).

### Lentiviral transduction and preparation of SK-Mel-28 cell lines stably transfected with DYRK3, p62-WT, p62-T269A, or p62-T269E

*S*K-Mel-28 cells stably overexpressing DYRK3, p62-WT, p62-T269A, or p62-T269E were generated by lentiviral transduction using gene-specific lentiviral vectors. Lentivirus particles were generated by co-transfection of 293T cells with lentiviral vectors and three plasmids (VSVG, RSV-REV, and PMDLg/pPRE) using the Lipofectamine PLUS reagent. Two days after transfection, the cell culture media were filtered using a 0.45 μm filter. SK-Mel-28 cells were then transfected with these lentiviral particles and selected using puromycin. The proper expressions of DYRK3, p62-WT, p62-T269A, and p62-T269E proteins were determined by Western blot analyses. As a negative control, a SK-Mel-28 cell line transduced with a scramble lentiviral vector was prepared and utilized.

### Wound healing assay

SK-Mel-28 transfected with either scrambled shRNA or shRNA-*DYRK3* were grown as a monolayer in 6-well plates subjected to a wound using a 1 ml micropipette tip. The cells were washed with serum-free DMEM to remove cell debris, incubated for 24 to 48 h in DMEM containing 10% FBS, and observed using light microscopy. The same assay was performed with SK-Mel-28 cells stably overexpressing DYRK3, p62-WT, p62-T269A, or p62-T269E, which were seeded cells into 6-well plates at the same density.

### Cell growth assay

Control SK-Mel-28 cells and SK-Mel-28 cells stably overexpressing DYRK3, p62-WT, p62-T269A, or p62-T269E were plated into 96-well plates at a density of 1 × 10^3^ cells per well and allowed to grow for 24, 48, and 72 h. The cell proliferation pattern was assessed at each time point using the cell counting kit-8. The same assay was performed with SK-Mel-28 cells stably transfected with shRNA-*DYRK3* or scrambled shRNA, following the same procedure.

### Colony formation assay

Control SK-Mel-28 cells or SK-Mel-28 cells stably transfected with DYRK3, p62-WT, p62-T269A, or p62-T269E were plated at a density of 500 cells per 60 mm plate and cultured for a duration of 14 days. Following the incubation period, the resulting colonies were stained using crystal violet. Subsequently, the identical assay was conducted using SK-Mel-28 cells stably transfected with either shRNA-DYRK3 or scrambled shRNA, following a similar procedure.

### Assessment of tumor growth for DYRK3- or p62-expressing SK-Mel-28 melanoma cells *in vivo*

Tumors were established in the abdomen of 6 week-old-male nude mice by subcutaneously injecting 1 × 10^7^ cells of SK-Mel-28 cells transfected with either an empty vector (mock) or SK-Mel-28 cell lines stably expressing different variants of DYRK3, p62-WT, p62-T269A, or p62-T269E. The tumor volumes were calculated using the major axis and minor axis measurements obtained with calipers and the following formula: tumor volume = (minor axis in mm)^2^ × (major axis in mm) × 0.523.

### Histological assessment of tumor tissues

Representative tumor sections from each group were stained with H&E and examined using a light microscope (Carl Zeiss Inc).

### Ethics approval of animal study

All animal facilities were approved by the Association for Assessment and Accreditation of Laboratory Animal Care. All animal experiments were approved by Animal Experimental Ethics Committee of Hanyang University and conducted according to the institutional guidelines established by the Hanyang University Institutional Animal Care and Use Committee.

### Statistical analyses

One-way ANOVA was used for all statistical analyses to compare data from different groups followed by Tukey’s post hoc test. The subsequent analysis was performed using SPSS statistical analysis software (version 26.0; IBM, https://www.ibm.com/kr-ko/spss). Values are reported as mean ± SD. The densities of Western blot bands were measured using GelQuant.NET software (version 1.8.2), following the protocol provided by the manufacturer (http://www.biochemlabsolutions.com).

## Data availability

The data used and/or analyzed during the current study are available from the corresponding author on reasonable requests.

## Supporting information

This article contains supporting information.

## Conflict of interest

The authors declare that they have no conflicts of interest with the contents of this article.
