# Supplementary material for: Dual-specificity kinase DYRK3 phosphorylates p62 at the Thr-269 residue and promotes melanoma progression
Source: J Biol Chem. 2024 Mar 20;300(4):107206. doi: 10.1016/j.jbc.2024.107206 (PMC11021969; doi:10.1016/j.jbc.2024.107206)
Supplement: Supporting Figures S1–S3 [file mmc1.pdf]

Supporting information

Dual-specificity kinase DYRK3 phosphorylates p62 at Thr-269 residue and promotes melanoma progression

Ye Hyung Lee, A-Rum Yoon, Chae-Ok Yun, and Kwang Chul Chung

Contents

1. **Figure S1.** DYRK3 phosphorylates p62 in the TB domain.
2. **Figure S2.** DYRK3 and p62 are colocalized within the melanoma cells.
3. **Figure S3.** Phosphorylation of p62 at T269 by DYRK3 promotes the activation of the mTORC1 pathway in melanoma cell.

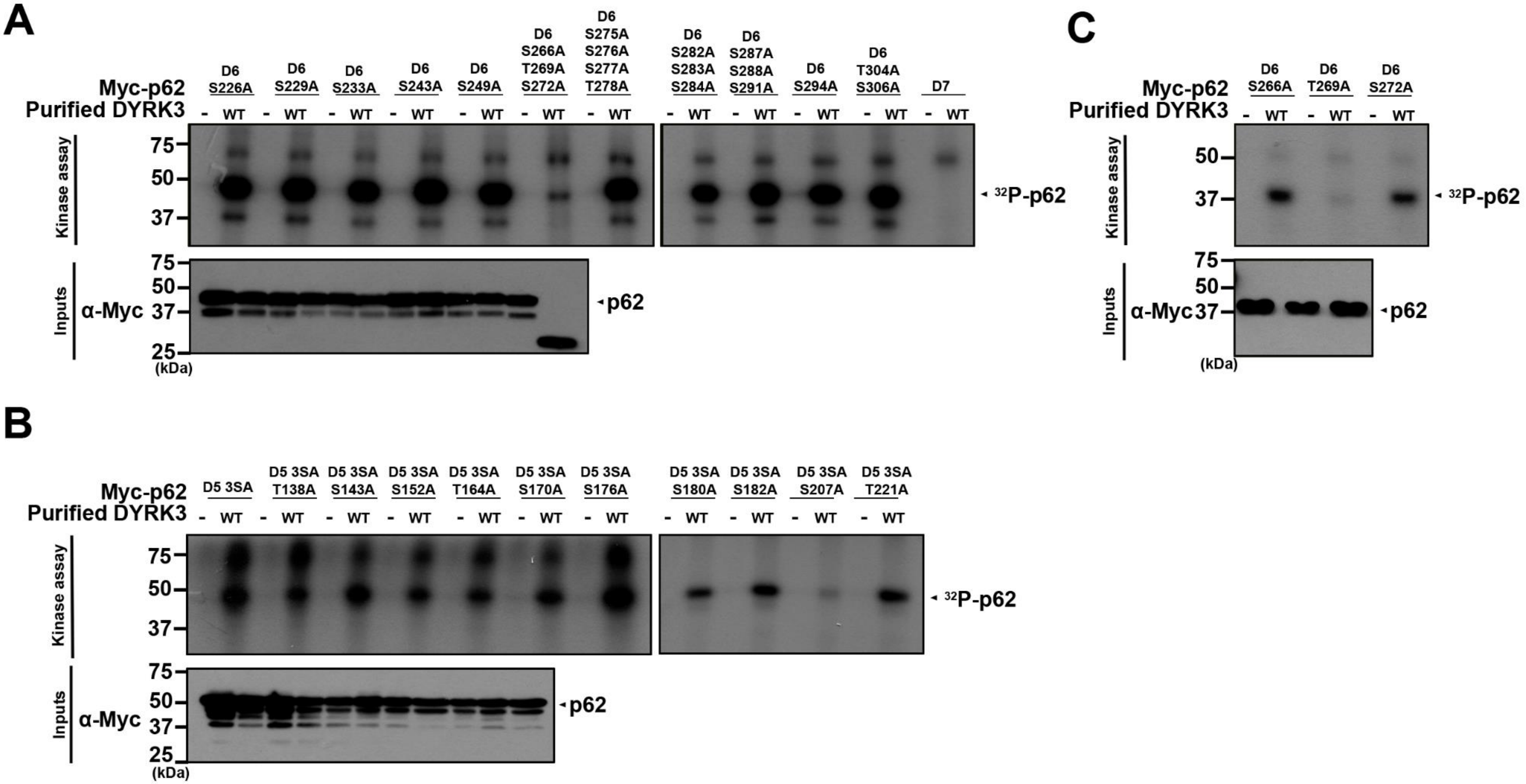

Figure S1. DYRK3 phosphorylates p62 in the TB domain. (A-C) Where indicated, HEK293 cells were transfected for 24 h with a plasmid encoding Myc-p62-WT or one of its point-mutants with single or double mutations at the specified Ser or Thr to Ala. Cell lysates (~1,000 μg of protein) were immunoprecipitated with an anti-Myc antibody. The samples were then mixed with bacterially expressed DYRK3-WT, incubated for 30 min at 30°C with the kinase buffer and [γ-^{32}P]ATP, resolved by SDS-PAGE, and analyzed by autoradiography. Proper expression of transiently expressed p62 in cell extracts was verified by immunoblotting with an anti-Myc antibody (Input).

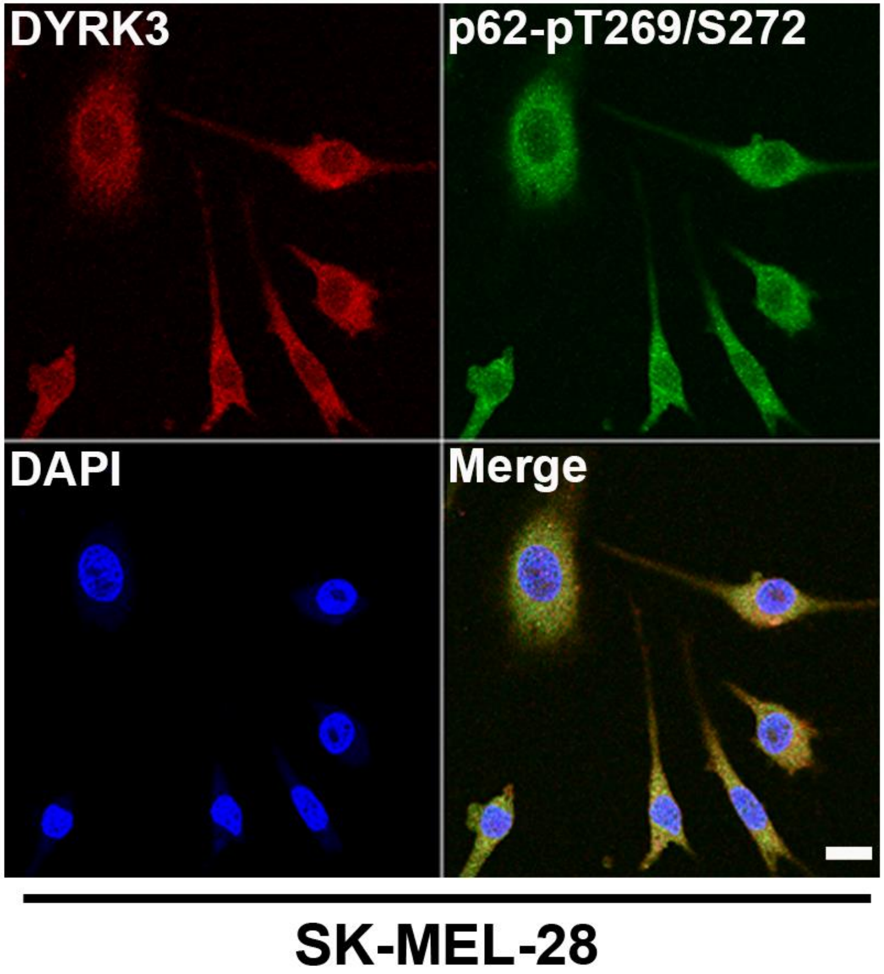

Figure S2. DYRK3 and p62 are colocalized within the melanoma cells. Representative confocal images of immunostaining of SK-Mel-28 cells with both anti-DYRK3 (red) and anti-p62-pT269/S272 (green) antibodies. Nuclei were counterstained with DAPI (blue). Scale bar = 20 μm.

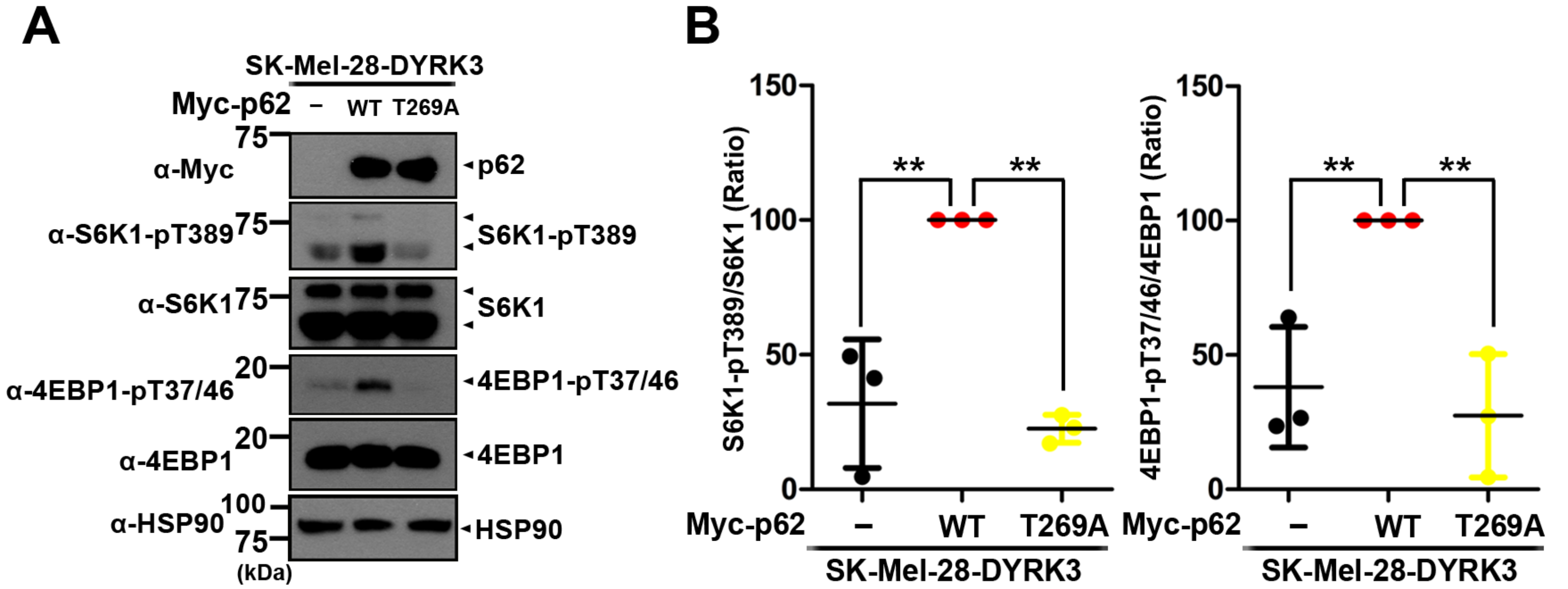

Figure S3. Phosphorylation of p62 at T269 by DYRK3 promotes the activation of the mTORC1 pathway in melanoma cell. (A-B) Where indicated, SK-Mel-28-DYRK3 cells were mock-transfected (-) or transfected for 24 h with a plasmid encoding Myc-p62-WT or Myc-p62-T269A, and subjected to western blotting with the indicated antibody. Data are represented as the mean ± standard deviation of three independent experiments ($**p < 0.01$)
